# Regulation of peptidoglycan synthesis by outer membrane proteins

**DOI:** 10.1016/j.cell.2010.11.038

**Published:** 2010-12-23

**Authors:** Athanasios Typas, Manuel Banzhaf, Bart van den Berg van Saparoea, Jolanda Verheul, Jacob Biboy, Robert J. Nichols, Matylda Zietek, Katrin Beilharz, Kai Kannenberg, Moritz von Rechenberg, Eefjan Breukink, Tanneke den Blaauwen, Carol A. Gross, Waldemar Vollmer

**Affiliations:** 1 Department of Microbiology & Immunology, University of California at San Francisco, San Francisco, CA 94158-2517, USA; 2 Centre for Bacterial Cell Biology, Institute for Cell and Molecular Biosciences, Newcastle University, Richardson Road, Newcastle upon Tyne, NE2 4AX, UK; 3 Molecular Cytology, Swammerdam Institute for Life Sciences, Faculty of Science, University of Amsterdam, Science Park 904, 1098 XH Amsterdam, The Netherlands; 4 Oral and Craniofacial Sciences Graduate Program, University of California at San Francisco, 513 Parnassus Ave, CA-94143, USA; 5 Mikrobielle Genetik, Universität Tübingen, Auf der Morgenstelle 28, 72076 Tübingen, Germany; 6 Prolexys Pharmaceuticals, Inc., 2150 West Dauntless Avenue, Salt Lake City, Utah 84116, USA; 7 Center of Biomembranes and Lipid Enzymology, Department of Biochemistry of Membranes, Institute for Biomembranes, University of Utrecht, Padualaan 8, 3584 CH Utrecht, The Netherlands; 8 Department of Cell and Tissue Biology, University of California at San Francisco, San Francisco, California 94158, USA

## Abstract

Growth of the meshlike peptidoglycan (PG) sacculus located between the bacterial inner and outer membranes (OM) is tightly regulated to ensure cellular integrity, maintain cell shape and orchestrate division. Cytoskeletal elements direct placement and activity of PG synthases from inside the cell but precise spatiotemporal control over this process is poorly understood. We demonstrate that PG synthases are also controlled from outside the sacculus. Two OM lipoproteins, LpoA and LpoB, are essential for the function respectively of PBP1A and PBP1B, the major *E. coli* bifunctional PG synthases. Each Lpo protein binds specifically to its cognate PBP and stimulates its transpeptidase activity, thereby facilitating attachment of new PG to the sacculus. LpoB shows partial septal localization and our data suggest that the LpoB-PBP1B complex contributes to OM constriction during cell division. LpoA/ LpoB and their PBP docking regions are restricted to γ-proteobacteria, providing models for niche-specific regulation of sacculus growth.

## Introduction

The stress-bearing peptidoglycan (PG) sacculus is essential for maintaining the shape and osmotic stability of almost all bacteria and its biosynthetic machinery is one of the most common targets of numerous antibiotics (Vollmer et al., 2008a). The net-like sacculus is made of glycan strands crosslinked by short peptides and forms a continuous layer surrounding the inner membrane (IM). Gram-positive bacteria have a multi-layered sacculus with covalently attached anionic cell wall polymers and cell surface proteins. In gram-negative bacteria, such as *E. coli*, the predominantly single-layered sacculus is firmly connected to the outer membrane (OM) by covalent and non-covalent interactions with various OM proteins. Enlarging this thin sacculus is a highly dynamic but poorly understood process. The PG layer must maintain structural integrity during a growth process that involves insertion/attachment of new glycan strands/patches and concomitant release of old material, also known as PG turnover (Park and Uehara, 2008). Additionally, PG synthesis and turnover must be spatially controlled to maintain cell shape, and temporally coordinated with the synthesis of other cell envelope layers for a successful cell cycle.

To generate and maintain proper morphology, rod-shaped bacteria engage in at least two different modes of PG synthesis (Vollmer and Bertsche, 2008). Small, newly divided cells exhibit a constant diameter and undertake an “elongation” mode of PG synthesis that increases the length of the lateral wall of the cell. As the cells grow longer, PG synthesis concentrates at midcell, eventually switching to a “constrictive” mode that allows cell division. Bacterial cytoskeletal proteins guide each of these processes (Shih and Rothfield, 2006). The bacterial actin homolog MreB is essential for elongation in many rod-shaped bacteria. Assisted by scaffolding and anchoring proteins (MreC, MreD, RodZ, RodA), MreB forms a membrane associated helical filament that positions and/or controls PG “elongasome” complexes along the sidewall to facilitate dispersive PG synthesis (Daniel and Errington, 2003). The bacterial structural homologue of tubulin, FtsZ, is required for PG synthesis at the septum. FtsZ forms a ring-structure at midcell. The “Z-ring” recruits 12 or more additional cell division proteins to form the dynamic, IM-localized divisome, which governs the synthesis of the two new poles of the daughter cells during cell division (Adams and Errington, 2009). FtsZ also drives a preseptal phase of cell elongation at midcell (Aaron et al., 2007; de Pedro et al., 1997).

MreB and FtsZ and their associated proteins nucleate an assemblage of IM localized or associated enzymes that make the PG building block and control PG synthesis. There is some specialization of the localization of PG synthases in *E. coli* (Vollmer and Bertsche, 2008). The essential PBP2 and PBP3 transpeptidases (TPases) are localized respectively at MreB or FtsZ sites. PBP1B, one of the two major bifunctional GTase (glycosyltransferases)-TPases (class A PBPs) is recruited to the divisome (Bertsche et al., 2006), whereas PBP1A has a preference for the sidewall of elongating cells (MB, BvdBvS, JV, TdB and WV, manuscript in preparation). However, PBP1A and PBP1B can substitute for each other, indicating that specificity is not complete (Yousif et al., 1985). In addition to many redundant synthases, bacteria also possess a large suite of PG hydrolases (amidases, endopeptidases, lytic tranglycosylases, carboxypeptidases; Vollmer et al., 2008b). Some of these PG hydrolases as well as their newly identified activators have been reported to localize at division sites in *E. coli* (Uehara et al., 2010) and it is likely that other hydrolases are present at MreB elongation sites, as is LytE in *B. subtilis* (Carballido-Lopez et al., 2006). It has been hypothesized that OM-anchored hydrolases form multi-enzyme complexes with IM-localized synthases to spatiotemporally coordinate their actions and provide safe enlargement of the sacculus and cell septation (Höltje, 1998). This model is supported by several interactions detected between PG enzymes (summarized in Vollmer and Bertsche, 2008), but direct evidence for such complexes is still missing. Gram-negative bacteria must also coordinate OM invagination with septal cleavage. Long thought to be a passive consequence of constriction, current work suggests that the 5-member Tol-Pal complex may facilitate OM invagination by a repeated sequence of events that first tether and then release OM-to-PG and OM-to-IM (Gerding et al., 2007). As Tol-Pal is not essential, other systems may also facilitate OM invagination.

The overall emerging picture is that PG synthesis is controlled both spatially and functionally by cytoskeletal elements from the inside of the cell, whereas hydrolysis is controlled from outside the sacculus. Our work challenges that view for Gram-negative bacteria. We identified two OM lipoproteins, LpoA and LpoB, which are absolutely required for the *in vivo* function of PBP1A and PBP1B, respectively. Each Lpo protein interacts specifically with its cognate PG synthase and stimulates its TPase *in vitro*. LpoB, like PBP1B, is recruited to the divisome but also to the lateral wall, whereas LpoA concentrates more at the sidewall of elongating cells. PBP1B/LpoB may also play a second role in division, working in tandem with the Tol-Pal complex to facilitate OM constriction. Moreover, we provide evidence that the Lpo proteins and their docking domains in PBPs show similar evolutionary distribution and are confined to the γ-proteobacteria. Modification of PG synthase activity in different bacterial groups might permit the lifestyle diversification necessary for expansion of ecological niches. *In toto*, our data indicate that in at least some gram-negative bacteria the enlargement of the PG layer requires control or activation of PG synthases not only from inside the cell (by the cytoskeleton) but also from outside by proteins associated with the OM. An independent parallel study by Paradis-Bleau et al. corroborates this notion.

## Results

### Identification of two novel PBP-interacting OM lipoproteins

**Figure 1:**
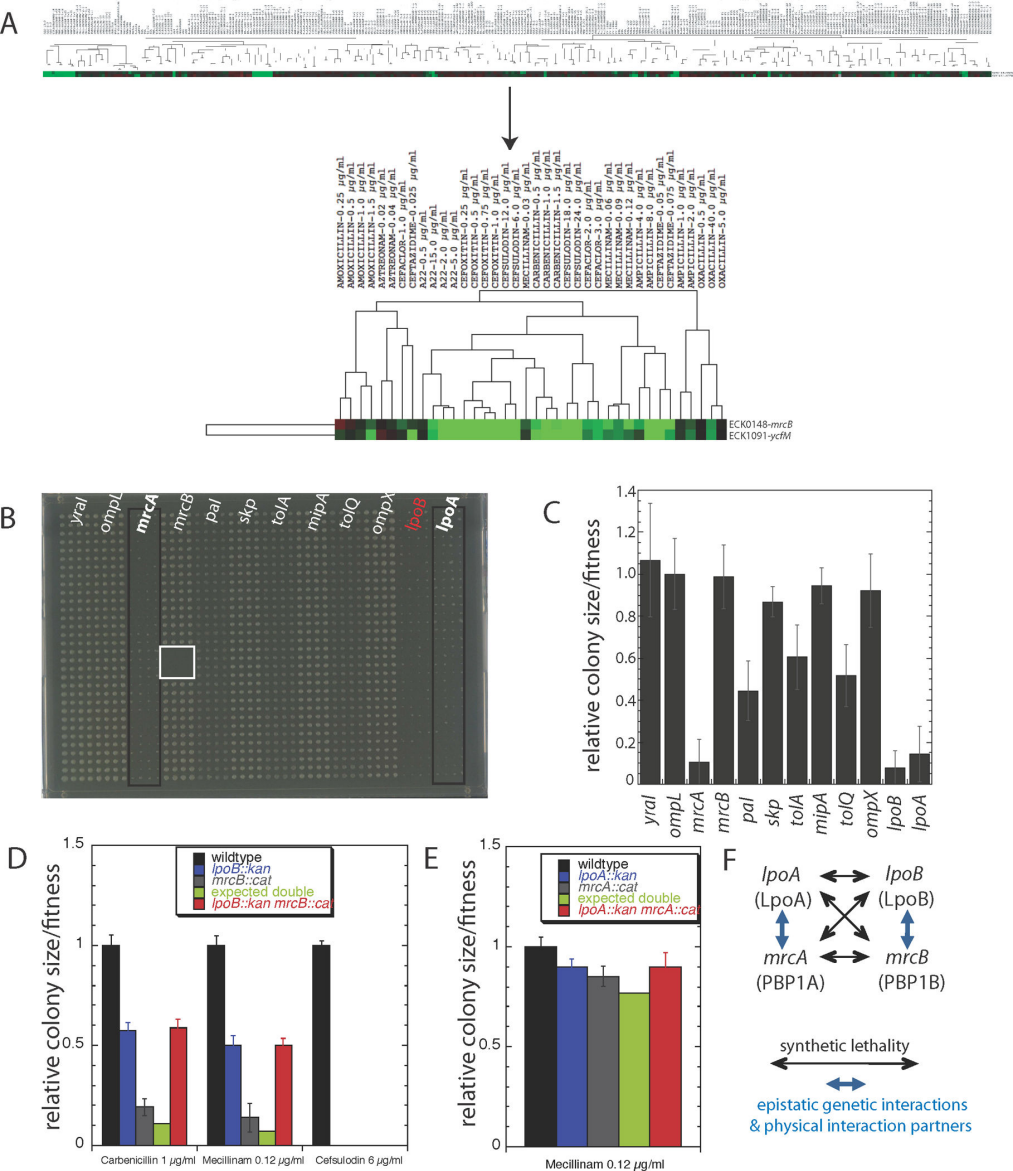
Identification of two OM lipoproteins that regulate the activity of the major *E. coli* PG synthases. **A.** The growth phenotypes of *lpoB*^−^ (*ycfM*) and *mrcB*^−^ cluster strongly across 324 different conditions (cc = 0.9; p<10^−116^). Cellular fitness is depicted using a color scale: red (increased); green (decreased) fitness. The upper panel illustrates that the highly correlated growth phenotypes of the two mutant strains depend on strong responses to only a few of the 324 conditions tested; the lower panel (blow-up) shows that these conditions are sub-lethal doses of β-lactams (target TPase domain of PBPs) and A22 (targets MreB). **B–C.** *lpoB*^−^ is synthetically lethal with both *mrcA*^−^ and *lpoA*^−^. Using high-throughput Hfr mating, we produced a 12 × 12 genetic interaction matrix. Results from pseudo-Hfr *lpoB::cat* crossed with 12 Kan^R^ recipients arrayed in 1536 format (boxes of 4 × 32 = 128 replicas) on LB are shown in (**B**) and quantified in (**C**). Recipients are indicated above the double mutant plate (**B**) and have colony sizes similar to the wildtype as single mutants (data not shown); the self mating control (*lpoB::cat* x *lpoB::kan*; red), demonstrates the low false-positive rate, since a double mutant of the same gene cannot be made in haploid organisms; the white box is a sterility control. *lpoB*^−^ is synthetically lethal with *mrcA*^−^ and *lpoA*^−^, and synthetically sick with deletions of all *tol*-*pal* components. The other 6 genetic interactions are neutral. Error bars depict standard deviations (n = 128). *lpoA*^−^ is synthetically lethal with both *mrcB*^−^ and *lpoB*^−^ (Fig. S1A–B). **D–E.** *lpoB*^−^ and *lpoA*^−^ show epistatic genetic interactions with *mrcB*^−^ (**D**) and *mrcA*^−^ (**E**) respectively. Quantifications of growth of wildtype, single mutant and the double mutant strains arrayed in 384-format (n = 96 colonies each) on LB agar plates containing different antibiotics (from Fig. S1C–F). Double mutant phenotypes are similar to single *lpo* mutant phenotypes indicating that each Lpo protein is absolutely required for the activity of its cognate PBP. **F.** Summary of genetic and physical interactions between Lpo proteins and PBP1A**–**PBP1B.

We employed two global approaches to identify proteins important for PBP1A and PBP1B function. First, as part of a broader chemical genomic screen (RJN, AT and CAG; manuscript submitted) we identified gene deletions whose phenotypes closely mirrored those exhibited by loss of PBP1B (*mrcB*^−^). The *E. coli* single gene knockout library was grown in sub-lethal concentrations of numerous drugs covering a broad spectrum of cellular targets, and environmental stresses reflecting the challenges *E. coli* faces in its natural environment. Analysis of the responses to all 324 conditions indicated that the growth phenotypes of *ycfM*^−^, encoding a putative OM lipoprotein, and *mrcB*^−^ were highly correlated (Fig. 1A top; correlation coefficient of 0.9, p<10^−116^), as a result of shared sensitivity to many β-lactams and to the MreB-specific inhibitor, A22 (Fig. 1A bottom). *ycfM*^−^ phenotypes were complemented by *in trans* expression of *ycfM* (data not shown). Second, we used a proteomic approach to identify novel interaction partners of PG synthases. Following application of a membrane fraction to agarose-bead coupled PBP1A or PBP1B, we identified one novel predicted OM lipoprotein with specific affinity for each PBP. YcfM was present only in the PBP1B eluate, whereas YraM was identified only in that from PBP1A (data not shown). Subsequent experiments confirmed that each PBP required its OM protein interaction partner for function. We renamed these proteins LpoA (YraM) and LpoB (YcfM) for Lipoprotein activator of PBP from the Outer membrane A & B.

### PBP1A or PBP1B activity in vivo is completely dependent on LpoA and LpoB

Although PBP1A and PBP1B have partially distinct roles in PG synthesis, the presence of one suffices for normal growth, but the absence of both PBPs (*mrcA*^−^*mrcB*^−^) leads to synthetic lethality despite the presence of a third, non-essential class A PBP (PBP1C) of unknown role. If LpoA and LpoB were essential for the function of their cognate PBP, then *lpoA*^−^ and *lpoB*^−^ should be synthetically lethal both with each other and with their non-cognate PBP, thereby mirroring the synthetic lethality of *mrcA*^−^ and *mrcB*^−^. We tested these and other double mutant phenotypes (Fig 1B,C & S1) using GIANT-coli, our recently developed high-throughput methodology for generating double mutants *en masse* (Typas et al., 2008). A 12 × 12 genetic interaction miniarray was generated by mating each Hfr donor (carrying a cat-marked gene deletion) to recipient cells (carrying kan-marked gene deletions) arrayed on agar plates; double mutant recombinants were selected by repinning cells onto double antibiotic plates. The double mutant growth phenotypes resulting from mating with pseudo Hfr *lpoB*^−^, displayed in Fig. 1B and quantified in Fig. 1C, reveal that in addition to synthetic lethality with *lpoA*^−^ and *mrcA*^−^*, lpoB*^−^ had specific negative interactions with gene deletions of the Tol-Pal system. We also quantified the genetic interaction of each *lpo* with its cognate PBP using drug conditions where the single mutants exhibited a partial growth defect, so the double mutant growth phenotypes could be accurately assessed. As expected for proteins working together, the double mutants exhibited epistatic interactions: removal of PBP in the absence of its cognate Lpo protein did not increase sensitivity to theβ-lactams tested (Fig. 1D,E & S1). *mrcB*^−^ cells grew worse than *lpoB*^−^ or *lpoB*^−^*mrcB*^−^ cells (Fig. 1D), suggesting that LpoB is deleterious in the absence of PBP1B, possibly due to additional interactions with other proteins (e.g. PG hydolases; see Discussion). The *in vivo* synthetic and epistatic interactions summarized in Fig. 1F indicate that LpoA/PBP1A and LpoB/PBP1B work together, and that each PBP absolutely requires its cognate Lpo protein for being functional *in vivo*.

### LpoA and LpoB are OM proteins, and interact both with PG and their cognate PBPs

Using specific antisera, we confirmed that LpoA and LpoB were located almost exclusively in purified OM rather than in IM vesicles (Fig. S2A), as predicted by their N-terminal signal peptide for lipid modification and OM sorting (Fig. S2B). Interestingly, both proteins interacted with isolated PG sacculi in a pull-down experiment (Fig. S2C). These results suggest that the Lpo proteins are OM attached lipoproteins that reach into the periplasm to interact with the PG layer.

**Figure 2:**
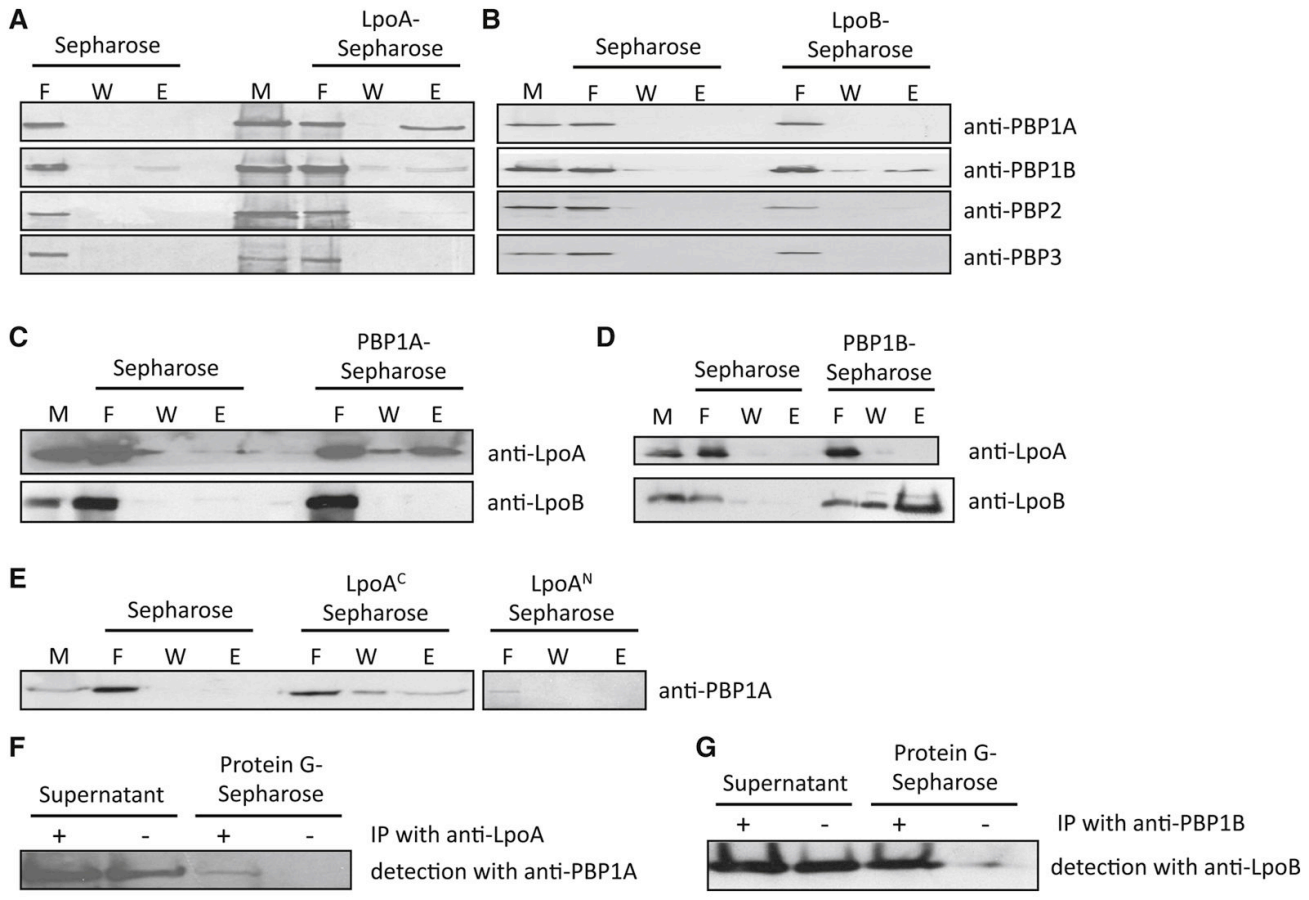
Each Lpo protein physically interacts with its cognate PG synthase *in vitro* and *in vivo*. LpoA specifically interacts with PBP1A (**A, C**), using its C-terminal domain (**E**); LpoB specifically interacts with PBP1B (**B**, **D**). Affinity chromatography with an *E. coli* membrane fraction applied to sepharose columns with different immobilized proteins; empty sepharose columns serve as controls. The membrane fraction (M) was applied to the columns in the presence of 400 mM NaCl to detect strong interactions and the flowthrough was collected (F). After washing (W), retained proteins were eluted with buffer containing 2 M NaCl (E). Samples were subjected to SDS-PAGE and Western blotting, followed by immunodetection of Lpo proteins or PBPs. Note that PBP1B has a slight nonspecific binding to the sepharose column (**A**). Lpo proteins also localize to the OM and interact with PG (Fig. S2). **F–G.** LpoA and LpoB interact with their cognate PBP *in vivo. In vivo* cross-linking of Lpo proteins with PBPs. *E. coli* cells were treated with DTSSP cross-linker, and membrane fractions were isolated and immunoprecipitated either with LpoA or PBP1B antibodies (+) or without antibodies (-). Samples were incubated with protein G-agarose beads, centrifuged, and the supernatant collected. The beads were washed and resuspended (protein G samples). Supernatant and protein G samples were boiled in buffer with reducing agent to revert the crosslinking, and eluates were subject to SDS-PAGE and Western Blotting, followed by immunodetection of PBP1A or LpoB.

To test whether the Lpo proteins interact specifically with PG synthases, we performed affinity chromatography under stringent conditions. An *E. coli* membrane fraction, which contains a large excess of other proteins over low-abundance PBPs, was applied at 400 mM NaCl to columns containing either immobilized LpoA or LpoB. PBP1A interacted only with LpoA, whereas PBP1B interacted specifically with LpoB (Fig. 2A,B). Conversely, LpoA and LpoB interacted with their immobilized cognate PBP (Fig. 2C,D), and the C-terminal domain of LpoA interacted with PBP1A (Fig. 2E). Importantly, we also detected LpoA-PBP1A and LpoB-PBP1B interactions in living cells with a crosslinking/ immunoprecipitation approach (Fig. 2F,G). Together, these results indicate direct interactions between LpoA and PBP1A, and between LpoB and PBP1B, confirming our genetic and chemical genetic inferences.

### Lpo proteins stimulate the TPase activity of their cognate PBP

**Figure 3:**
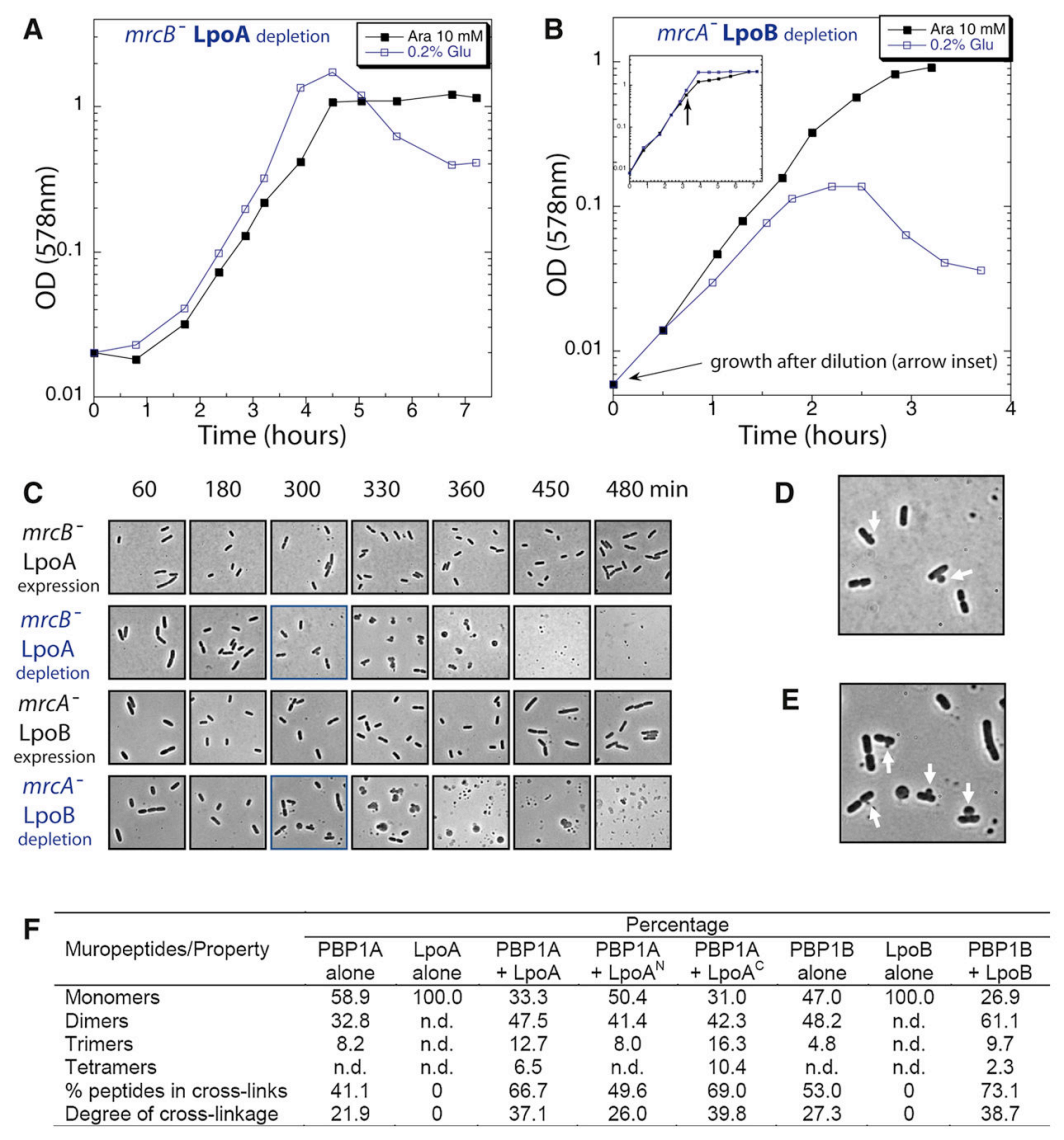
LpoA and LpoB are absolutely required for the *in vivo* function of their cognate PBP and strongly stimulate the TPase activity of their cognate PBP *in vitro*. **A–B.** Depletion of Lpo proteins in the absence of the non-cognate PBP leads to lysis. LpoA (**A**) and LpoB (**B**) were expressed from an arabinose (Ara)-inducible plasmid in *mrcB*^−^ and *mrcA*^−^ cells respectively, and depleted by dilution of stationary phase cultures into glucose-containing LB medium (repression). For LpoB-depletion, diluted cultures were first grown to OD_578_=0.6 in glucose LB medium (**B**, blue line, inset) and then rediluted into fresh glucose LB medium to observe lysis. **C–E.** Morphology of Lpo-depleted cells. Cells grown with glucose to deplete LpoA (in *mrcB*^−^ background) and LpoB (in *mrcA*^−^ background), or with Ara (control), were fixed and examined by phase contrast microscopy. Lysis of LpoA- or LpoB-depleted cells began after 300 min of growth in glucose. Magnified pictures of LpoA- (**D**) or LpoB-depleted (**E**) cells at 300 min reveal the presence of lysis bulges often emerging at midcell (arrows). (**F**) The activity of detergent-solubilized PBP1A or PBP1B was assayed with radiolabelled lipid II in the presence or absence of their cognate Lpo protein. The PG product was digested with cellosyl and the resulting muropeptides were analysed by HPLC (for chromatograms see Fig. S3). The table shows a summary of the types of muropeptides and properties of the PG synthesized. The % peptides in cross-links was calculated as 100% − % Monomers; the degree of cross-linkage is defined as %Dimers/2 + %Trimers × 2/3 + %Tetramers × 3/4 and is equal to the percent peptides that were used as donors in TPase reactions; n.d., not detected. Both Lpo proteins increased the cross-linkage in the PG synthesized by their cognate PBP. LpoA also stimulated the PBP1A-catalysed attachment of newly made PG to sacculi (Fig. S4).

We monitored the effects of depleting either LpoA or LpoB in cells lacking the non-cognate PBP (*mrcB*^−^ or *mrcA*^−^ respectively), by placing each *lpo* under the tightly controlled arabinose promoter. Cell lysis was observed upon Lpo depletion (Fig. 3A,B), and confirmed by phase contrast microscopy (Fig. 3C). Moreover, lysis was accompanied by formation of bulges at the cellular periphery, often at or near the midcell division sites (Fig. 3D,E), which appear similar to those seen upon treatment with PBP inhibitors like penicillin (Chung et al., 2009) or overexpression of catalytically inactive versions of PBP1B (Meisel et al., 2003). These cellular morphologies and the sensitivity of *lpoB*^−^ to numerous β-lactams that target the TPase domain of active PG synthases, suggested that Lpo proteins might stimulate the TPase activity of their cognate PBP.

To test the hypothesis that Lpo proteins stimulate the activities of their cognate PBPs, we directly probed the enzymatic consequences of Lpo association with PBPs with a recently developed in vitro PG synthesis assay that uses radioactively labelled lipid II as a substrate and purified PBP1A or PBP1B (Bertsche et al., 2005; Born et al., 2006) with or without their cognate Lpo. HPLC analysis of the muramidase digested PG product allowed detection and quantification of both monomeric (uncrosslinked) and multimeric (crosslinked) products of the GTase and TPase activities of these PBPs (Fig. S3A). Although PBP1B and PBP1A themselves are highly active, each cognate Lpo enhanced transpeptidation (Fig. 3F & S3B). LpoB increased the percentage of cross-linked peptides in the PBP1B product from 53 to 73%, whereas LpoA increased the crosslinkage in the PBP1A product from 41 to 67%. The C-terminal domain of LpoA (LpoA^C^) alone stimulated the TPase activity of PBP1A (Fig. 3F) consistent with its interaction with the enzyme (Fig. 2E). The cognate Lpo proteins stimulate PBP1A and PBP1B to produce not only dimeric but also trimeric and tetrameric structures in which 3 and 4 peptides are connected (Fig. S3C). Although tetrameric peptides exist in isolated sacculi, they have never been observed in PBP reactions *in vitro*. Finally, using a separate assay, we found that LpoA, but not the truncated LpoA^C^ or LpoA^N^ stimulated the capacity of PBP1A to attach *in vitro* synthesized, new PG to sacculi by transpeptidation reactions from 44% to 66% (p = 0.057, Fig. S4). Thus each Lpo stimulates the TPase activity of its cognate PBP.

### Lpo proteins localize to the sidewall and septum independently from their cognate PBPs, but septal localization is dependent on FtsZ, FtsI (PBP3) and ongoing PG septal synthesis

**Figure 4:**
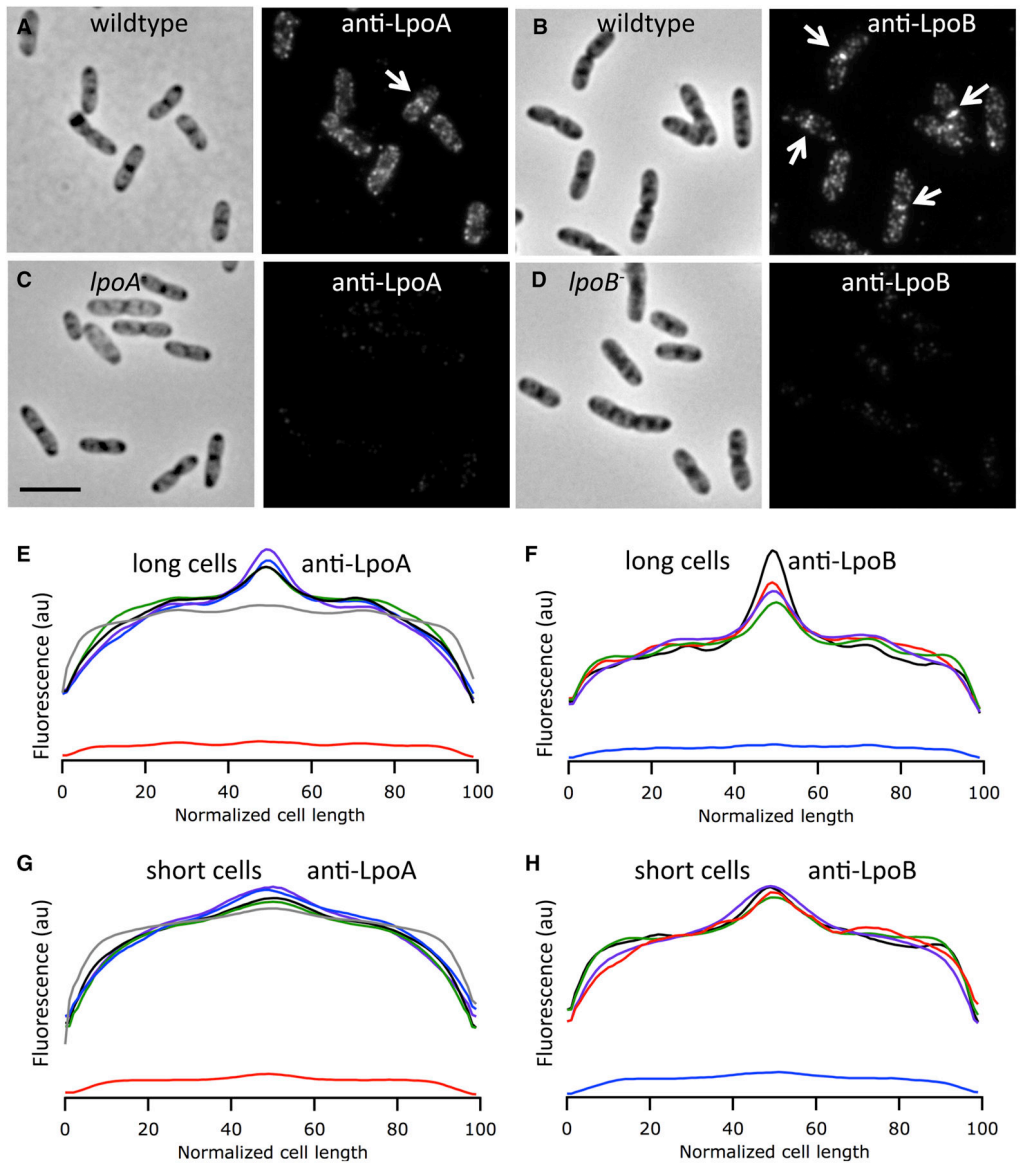
LpoA and LpoB localize as distinct foci in the lateral wall and at constriction sites of dividing cells. *E. coli* wildtype (TB28) (**A**) and its *lpoA*^−^ derivative (**C**) were immunolabeled with antibodies against LpoA. *E. coli* wildtype (BW25113) (**B**) and its *lpoB*^−^ derivative (**D**) were immunolabeled with affinity-purified antibodies against LpoB. The immunolocalization procedure does not affect the cell membrane (Fig. S5A) or the size/shape of the cells (Supplemental Experimental Procedures). The left side of each dual panel shows the phase contrast image and the right side the corresponding fluorescence image. The scale bar equals 5 μm. Arrows in panels A & **B** depict LpoA and LpoB foci for cells engaged in septation. Panels **E–H** show the average LpoA (**E** & **G**) or LpoB (**F** & **H**) fluorescence intensity profiles of >1000 individual cells per strain plotted against the relative position along the length axis of the cell. The populations of cells were split into longer cells (1/3 of the population), enriched in dividing cells (**E** & **F**) and shorter cells (2/3 of the population), including only few dividing cells (**G** & **H**). For panels **E–H**: black lines: wildtype cells; red lines: *lpoA*^−^ cells; blue lines: *lpoB*^−^ cells; green lines: *mrcA*^−^ cells (lacking PBP1A) and purple lines: *mrcB*^−^ cells (lacking PBP1B). The grey line in panels **E** & **G** are from a general membrane staining using BODIPY 558/568 C12. LpoB localizes late in the cell cycle to midcell (Fig. S5B). Midcell localization of LpoB depends on the presence of FtsZ, PBP3 and ongoing septal PG synthesis (Fig. S6).

We used immunolabeling and fluorescence microscopy to detect the position of LpoA and LpoB, employing a non-perturbing protocol for fixing cells and permeabilizing their OM and PG (see Fig. S5A and Supplementary Experimental procedures). The data are displayed both as representative single cell images (Fig. 4A-D) and as fluorescence profiles across >1000 size selected cells (Fig. 4E–H). The low background signal in the absence of the cognate protein shown by examination of images (Fig. 4C,D), quantitative analysis (Fig. 4E–H) and Western blot analysis (data not shown) indicated that both primary antibodies were specific. LpoA and LpoB were each detected as foci in the peripheral part of the cell, with LpoB, and to a lower degree LpoA, also exhibiting relatively intense labeling at the midcell of dividing cells (Fig. 4A,B). The quantified fluorescence intensity profiles validated our qualitative observations, and further established that LpoB, and to a lower degree LpoA, have stronger midcell labeling intensity in the longer (i.e. dividing) cells (Fig. 4E,F) than in the shorter cells (Fig. 4G,H). The localization of both Lpo proteins was maintained in the absence of the cognate/non-cognate PBP and in the absence of the other Lpo protein (Fig. 4E–H), indicating that LpoA/B localize independently of these proteins. Immunoblot analysis indicated that cellular amounts of LpoA and LpoB remained constant in all mutants (data not shown).

The fluorescence profiles of LpoA and LpoB in cells of different length classes indicated that localization to the septum began at 60% of the cell cycle (Fig. S5). As this coincides with proteins that localize in the second step in divisome maturation (Aarsman et al., 2005), localization of Lpo proteins might depend on FtsZ and/or FtsI (PBP3). Indeed, in the FtsZ temperature sensitive strain *ftsZ84*(ts), the LpoB midcell localization observed at 28 C was abolished two mass doublings after shift to the non-permissive temperature of 42°C (Fig. S6D–F). Likewise, midcell localization of LpoB was abolished when a strain expressing the temperature sensitive variant of PBP3 *ftsI2158*(ts) was shifted to 42°C for two mass doublings (Fig. S6J–L). On the other hand, LpoA was poorly localized overall in the FtsZ(ts) and PBP3(ts) strains (Fig. S6A–C & G–I). This phenotype is consistent with the weaker midcell localization of LpoA in wildtype cells (Fig. 4A). At the non-permissive temperature, PBP3(ts) cells filament and have blunt constrictions where septation would normally occur. Neither LpoA nor LpoB localized at these constrictions (Fig. S6H, I, K & L). To address whether ongoing septal PG synthesis is the cue for LpoB localization, we specifically inhibited PBP3, the TPase essential for septal PG synthesis, with aztreonam and observed that LpoB lost its septal localization after 45 min of drug treatment (Fig. S6M–O), whereas PBP3, one of the late divisome members, still localized at the septum (data not shown). In summary, LpoB is likely to require ongoing septal PG synthesis for midcell localization, whereas LpoA localizes predominantly to the lateral wall.

### A secondary role for LpoB/PBP1B in OM constriction during cell division

The Tol-Pal complex is pivotal for envelope integrity. Mutants in this complex exhibit periplasmic leakage, increased vesicle formation and sensitivity to many drugs (Bernadac et al., 1998; Cascales et al., 2002). It was recently proposed that by localizing at constriction sites and alternately tethering the OM to PG or to the IM, Tol-Pal may synchronize invagination of the OM with constriction of the IM and PG layers during cell division (Gerding et al., 2007). Given the importance of this function, it was surprising that members of the Tol-Pal complex are not essential in *E. coli*, suggesting the possibility that back-up systems also perform this function. Interestingly, the LpoB-PBP1B transenvelope complex, like the Tol-Pal complex, can tether the OM either to the PG (LpoB-PG interaction; Fig. S2C) or to the IM (LpoB-PBP1B interaction; Fig. 2). Moreover, LpoB-PBP1B, like Tol-Pal, localizes at constriction sites (Fig. 4, Bertsche et al., 2006) and both *lpoB*^−^ and *mrcB*^−^ were synthetically sick in combination with *tol*-*pal* mutants (Fig. 1B,C and data not shown). In contrast *lpoA*^−^ or *mrcA*^−^ exhibited only marginal genetic interactions with *tol*-*pal* mutants (see Fig. S1 and its legend).

**Figure 5:**
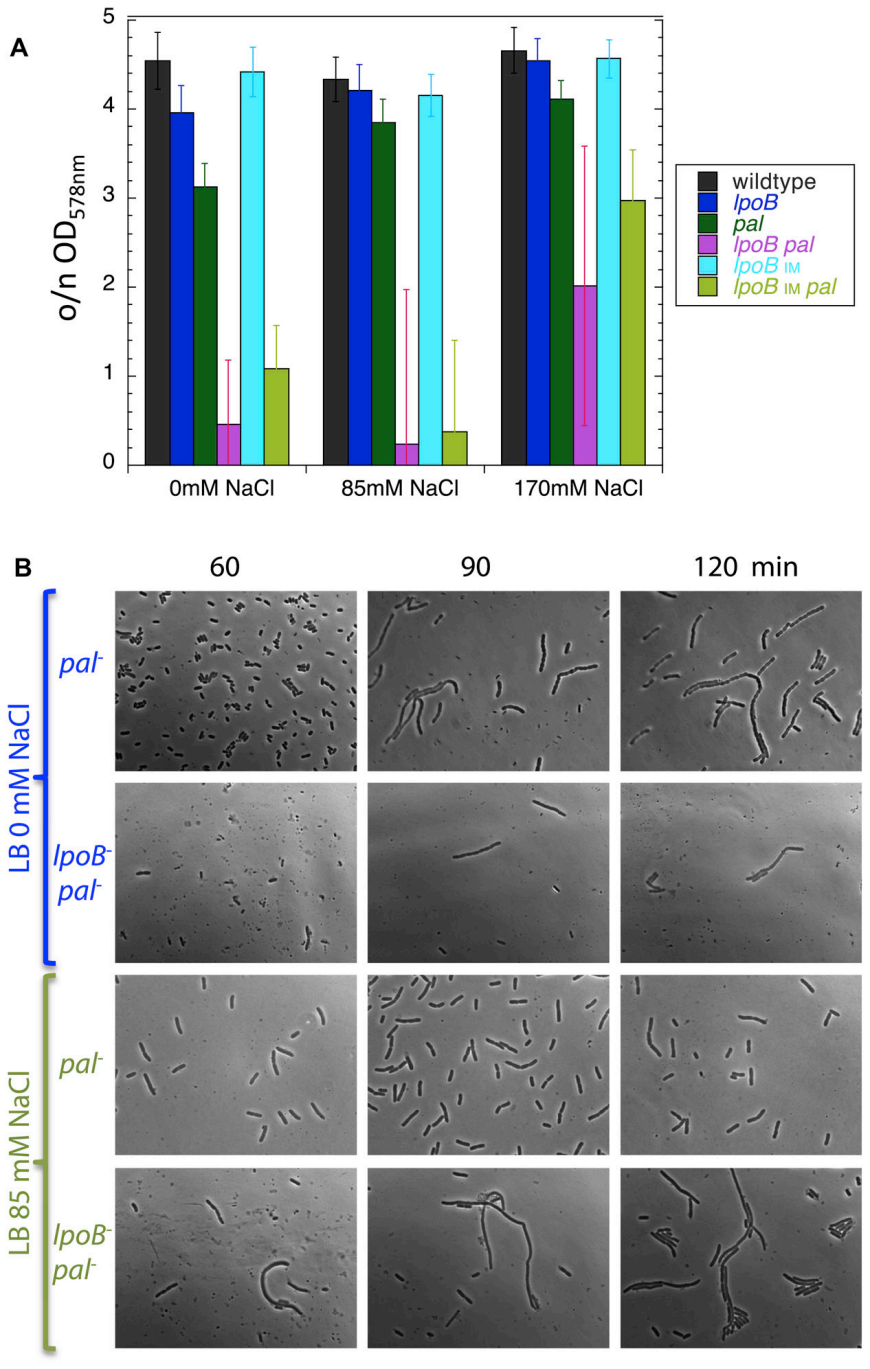
LpoB-PBP1B has a secondary role in OM invagination during cell division. **A.** OD_578_ of various strains measured after overnight growth (o/n) in LB with different amounts of salt. *lpoB*_IM_ indicates an IM-localized variant of LpoB, created by changing its lipoprotein sorting signal. Lysis phenotypes of *lpoB*^−^*pal*^−^ and *lpoB*_IM_*pal*^−^ cells are indistinguishable and are synthetic when compared to the lysis patterns of the individual single mutants. Error bars are based on n>6 repetitions of the growth experiments. The large error bars for *lpoB*^−^*pal*^−^ and *lpoB*_IM_*pal*^−^ are likely due to suppressors arising at different time points during the slow growth and continuous lysis of these mutants at low salt concentrations, as all biological repetitions exhibited significant cellular debris, independent of the overnight OD_578_. Fig. S7 demonstrates that LpoB_IM_ was still able to partially activate PBP1B as it sustained viability in cells lacking either PBP1A or LpoA in LB no/low salt. **B.** Cellular morphologies of *pal*^−^ and *lpoB*^−^*pal*^−^ cells in LB containing no or low salt. Cells grown overnight in LB Miller (170 mM NaCl) were inoculated in LB containing no or low salt to an OD of 0.02, and then fixed and examined by phase contrast microscopy at regular intervals thereafter.

To further explore whether LpoB/PBP1B and Tol/Pal have partially redundant roles in OM constriction, we examined the phenotype of *lpoB*^−^*pal*^−^ cells in LB no salt conditions where the *pal*^−^ defect in cell division is manifest (Gerding et al., 2007), and also in LB low salt (85 mM). Under these conditions, *lpoB*^−^ and *pal*^−^ were synthetically lethal and *lpoB*^−^*pal*^−^ cells showed severe lysis after overnight growth, whereas each single mutant grew robustly and exhibited no significant lysis (Fig. 5A). We asked whether OM-localization of LpoB is important for complementing Tol-Pal function. An IM-localized LpoB (*lpoB*_IM_; created by changing the lipoprotein sorting signal of the chromosomal copy of *lpoB*) was almost as defective as *lpoB*^−^ in complementing *pal*^−^ mutants. *lpoB*_IM_*pal*^−^ cells lysed as severely as *lpoB*^−^*pal*^−^ cells after overnight growth in low salt (Fig. 5A). In stark contrast, LpoB_IM_ was still able to at least partially activate PBP1B as it could sustain viability in cells lacking either PBP1A or LpoA in LB no/low salt (Fig. S7).

In complementary studies, we compared *pal*^−^ and *lpoB*^−^*pal*^−^ cells morphologically after shift to either LB no or low salt (Fig. 5B); *lpoB*^−^ cells were also tested but did not show significantly stronger lysis or division defects than wildtype cells, and are not shown here. Although all cells appeared relatively healthy prior to shift (data not shown), by 60 min after shift to no salt, *lpoB*^−^*pal*^−^ cultures exhibited extensive lysis, whereas *pal*^−^ cultures did not. Examination of cell morphology at 85 mM NaCl (where more *lpoB*^−^*pal*^−^ cells survived) revealed that *lpoB*^−^*pal*^−^ cells had much more severe division defects than *pal*^−^ cells. Whereas *pal*^−^ cells formed only a few short chains with deeply constricted “individual” cells, *lpoB*^−^*pal*^−^ cells built long filaments with almost no constrictions, suggestive of an accumulated defect in cell division. In summary, the LpoB-PBP1B complex has all of the hallmarks of a machine that promotes OM constriction during cell division when Tol-Pal is absent. Our data also provide an explanation why Tol-Pal is not essential in *E. coli* even though its role is essential for cell proliferation.

### Both Lpo proteins and their interaction domains have recently evolved

**Figure 6:**
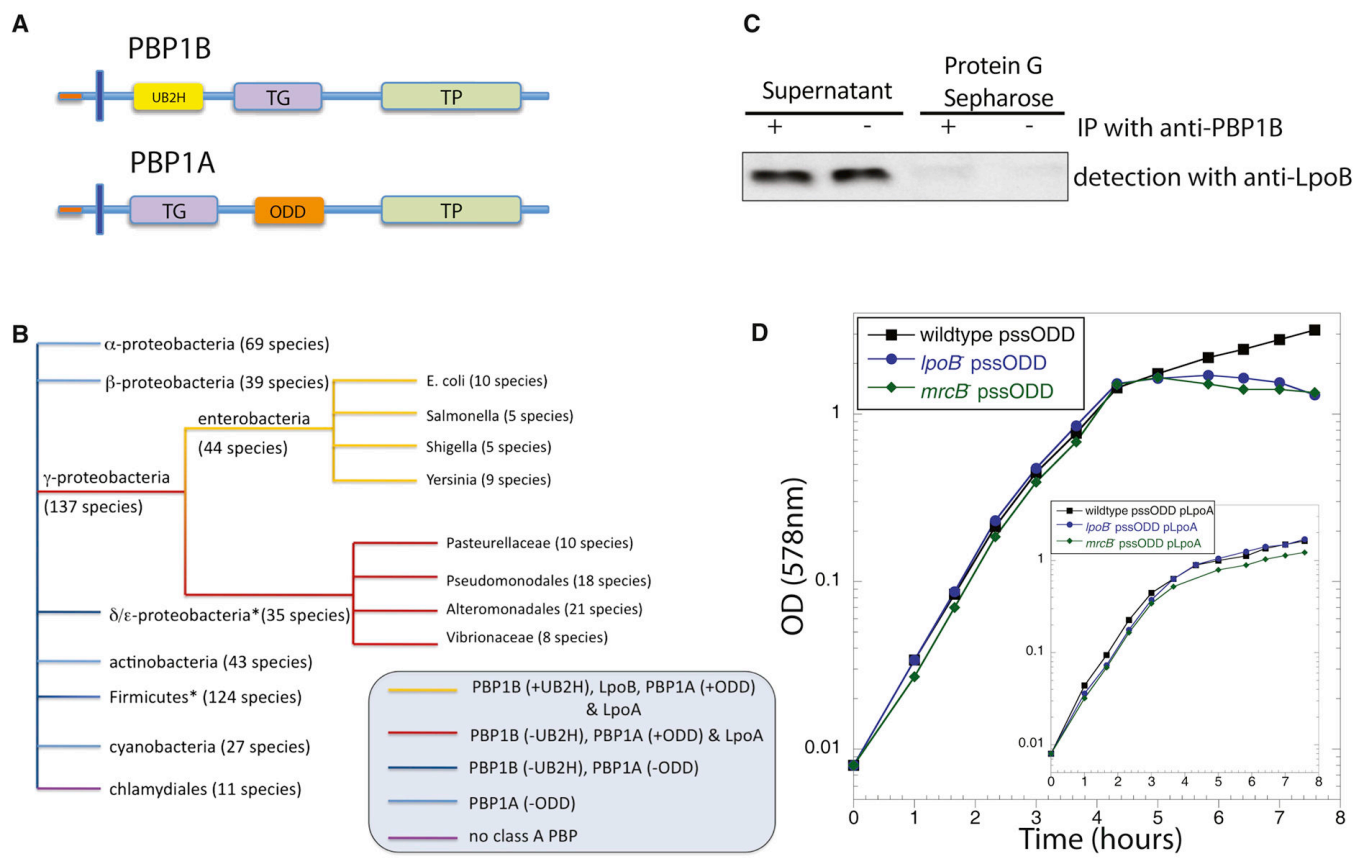
LpoA/LpoB and their docking domains in PBP1A/PBP1B have recently evolved together. **A.** Schematic representation of PBP1A and PBP1B, illustrating the conserved TPase and TGase domains of both proteins, as well as the newly evolved UB2H domain in PBP1B and the comparably sized insertion region ODD in PBP1A. **B.** Phylogenetic distribution of Lpo proteins and PBP1A/PBP1B with or without the docking regions. STRING (Jensen et al., 2009) was used for assessing protein and domain conservation over >400 bacterial species. ODD and LpoA are limited to γ-proteobacteria (red and yellow lines) and UB2H and LpoB are further restricted to enterobacteria (yellow lines); stringent cutoffs were used to assess conservation of LpoA and LpoB (100 bits), and of UB2H and ODD domains within the class A PBPs (35% amino acid sequence identity). Note that exceptions exist for some large bacterial clades depicted here; for example in the Firmicutes phylum, Mycoplasmae and Ureoplasma have no class A PBP, whereas staphylococci have only one class A PBP that has similar levels of homology to PBP1A and PBP1B. **C.** UB2H is the PBP1B docking domain of LpoB. LpoB does not interact with a PBP1B variant that lacks the UB2H (PBP1BΔUB2H). *In vivo* cross-linking/co-immunoprecipitation of LpoB with anti-PBP1B was performed as in Fig. 2G. **D.** ODD is the PBP1A docking region of LpoA. Overexpression of ODD with an N-terminal signal sequence for periplasmic localization (pssODD) leads to lysis in cells that depend on a functional PBP1A-LpoA complex [*mrcB*^−^ (green diamonds) and *lpoB*^−^ (blue circles)], but does not affect wildtype cells (black squares). Note that the OD axis is in log_10_ and there is a ~25% drop in cell culture density for *mrcB*^−^ and *lpoB*^−^ cells, leading to clear formation of cellular debris. Overexpression of LpoA together with pssODD averts lysis (inset panel).

PBP1A and PBP1B proteins have orthologues across all bacterial phyla with a cell wall whereas LpoA and LpoB are evolutionarily restricted to γ-proteobacteria and enterobacteria respectively. We considered the possibility that, like the Lpo-proteins themselves, the PBP domains interacting with each Lpo might have also arisen recently. Interestingly, *E. coli* PBP1B has an extra domain, UB2H (Sung et al., 2009), not present in *S. aureus* PBP2 (Lovering et al., 2007), and, like LpoB, this domain is strongly conserved only in the enterobacteria (Fig. 6B; yellow line). Likewise, a BLAST search revealed a region of PBP1A, comparable in size to UB2H, located between its TPase and GTase domains, which is present only in γ-proteobacteria (Fig. 6A,B; yellow and red lines) as is the case for LpoA.

We tested whether these domains, present in the same bacteria as their respective Lpo proteins, serve as their docking regions. For PBP1B, using available structural information, we constructed a chromosomal PBP1B variant without UB2H domain. Although this variant had been reported to provide a partially active PBP1B when significantly overexpressed (Sung et al., 2009), we found that neither endogenous expression nor overexpression of the variant complemented *mrcB*^−^ (data not shown). Importantly, the stable PBP1BΔUB2H was unable to crosslink with LpoB (Fig. 6C), consistent with the idea that UB2H interacts with LpoB, and that the reason for dysfunction of PBP1BΔUB2H is its inability to interact with LpoB. Lacking structural data for PBP1A we were unable to perform a comparable experiment. Instead, we pursued a strong prediction of the idea that the newly evolved PBP1A region is a docking domain for LpoA. Knowing that LpoA binds to PBP1A (Fig. 2) and is essential for PBP1A function (Fig. 3), we predicted that overexpressing this domain (ODD, for Outer membrane PBP1A Docking Domain) would titrate out LpoA and lead to lysis in cells lacking the PBP1B/LpoB pathway. Indeed overexpressing ODD fused to an N-terminal signal sequence did result in ~ 25% lysis as the culture density decreased (Fig. 6D) and cellular debris was clearly visible. This was a direct result of titrating LpoA away from PBP1A because lysis was averted when LpoA was coexpressed along with ODD (inset Fig. 6D). In summary, γ-proteobacteria have superimposed novel regulation on a broadly conserved biological process -PG synthesis- by coevolving interacting proteins and their PBP docking domains.

## Discussion

In the present report, we have identified two OM lipoprotein modulators, LpoA and LpoB, of the two major PG synthases in *E. coli*, PBP1A and PBP1B. Each Lpo protein is essential for the function of its cognate PBP synthase *in vivo*, and enhances its TPase activity *in vitro*. Moreover, the LpoB-PBP1B complex has a secondary role in OM constriction during cell division. LpoA and LpoB are unrelated in sequence and narrowly distributed in bacteria, and their interaction domains in the cognate PBP show similar distributions to the modulators themselves. Below we consider the implications of these findings.

### Modulation of PG synthases by OM proteins

**Figure 7:**
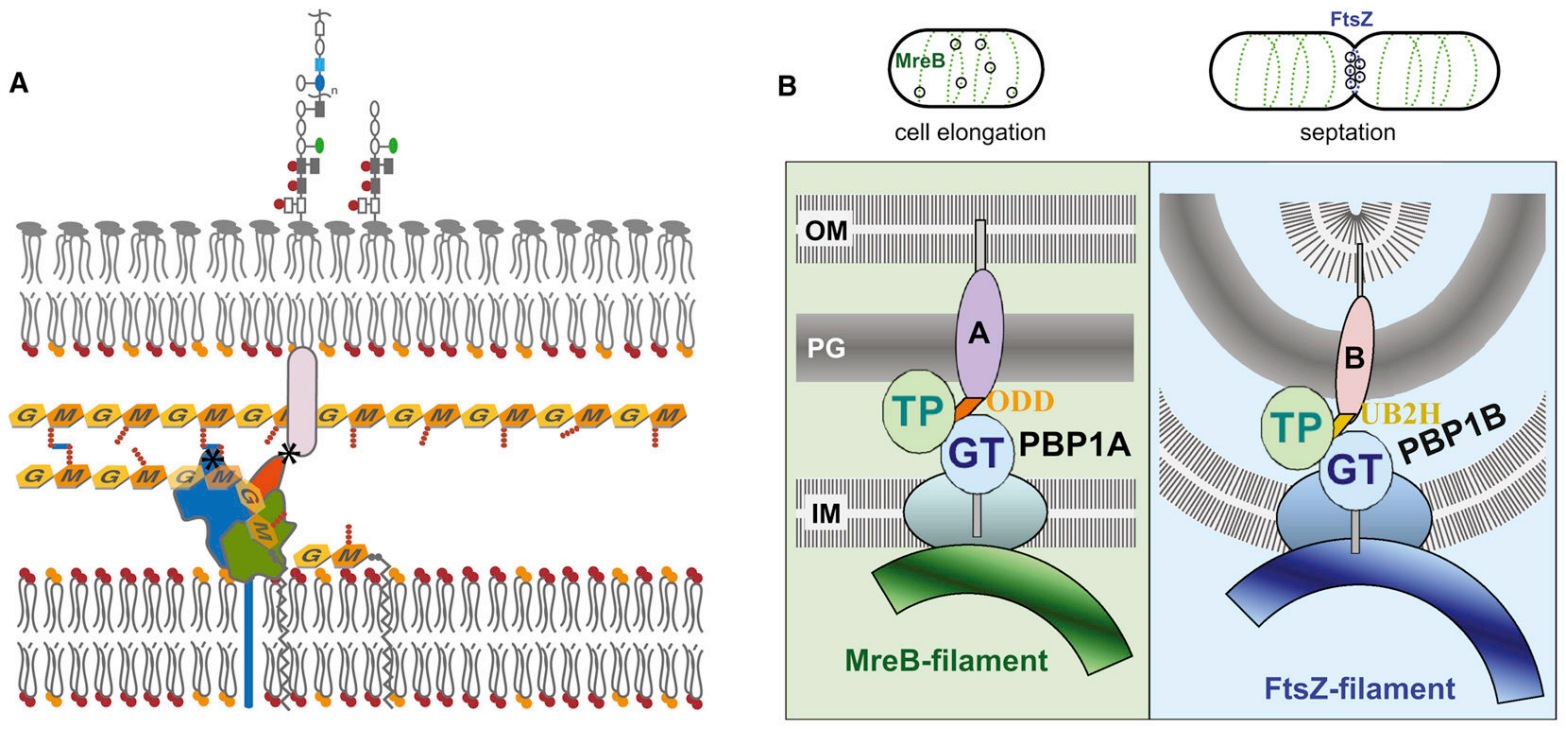
Model for the mechanism of action of Lpo proteins. **A.** The docking domain of the PBP interacts with its cognate Lpo, and undergoes a conformational change that repositions its TPase domain so that peptide crosslinking is stimulated. Glycan chains are sandwiched between the IM and OM, and are composed of N-acetylglucosamine (G) and N-acetylmuramic acid (M) depicted as hexagons. Attached to the M sugar are short peptides (balls) that crosslink the glycan strands. The 3-domain PBP is anchored to the IM [blue:TPase; green:GTase; orange: docking domain (UB2H/ODD)], and the Lpo protein (cylinder) is anchored to the OM. **B.** PBP1A-LpoA & PBP1B-LpoB are primarily responsible respectively for sidewall and septal PG synthesis. Cytoskeletal elements and the large elongasome/divisome complexes assembled around them recruit PBP1A at the lateral wall of elongating cells and PBP1B at septa of dividing cells. Here, IM components of these complexes are depicted as colored ovals, and periplasmic/OM components, including PG hydrolases and other PBPs, are omitted for clarity. LpoA and LpoB mirror the localization of their cognate PBP. Lpo proteins localize independently of their cognate PBP possibly via interaction with newly synthesized PG and/or via yet unidentified interactions to elongasome/divisome members. Despite their localization preferences, each PBP-Lpo complex can substitute for the loss of the other, which is reflected by the presence of both as foci at the lateral wall of cells and also at midcell of dividing cells. The docking domains for PBP1A (ODD) and PB1B (UB2H) are depicted here in orange and gold respectively.

Our work overturns the prevalent thinking that PG synthesis is controlled exclusively from inside the cell. It had been known that the bifunctional PBPs are recruited and positioned via interaction with IM-associated cytoskeletal complexes, which may also stimulate the GTase domain to synthesize the glycan strands (Uehara and Park, 2008). Here we show that some gram-negative bacteria also control PG synthesis from the outside of the sacculus, a regulatory strategy that may enable better coordination between PG growth and the two membranes that sandwich the sacculus. Upon direct interaction with the OM Lpo proteins, the TPase domain of each PBP is stimulated to form peptide cross-links during PG synthesis (Fig. 7A). The specific molecular mechanism by which Lpo proteins stimulate the peptide cross-linking activity of their cognate PBP remains to be determined. For example, interaction with Lpo could induce a conformational switch that repositions the TPase domain and affects acceptor peptide binding, attachment to the PG or the TPase activity itself. Concurrent work from Paradis-Bleau et al. suggests that one of the two Lpo proteins, LpoB exerts a small increase in the GTase rate of PBP1B.

A critical question is why PBP1A and PBP1B are completely dependent respectively on LpoA and LpoB for function *in vivo*, when both synthesize a crosslinked PG from lipid II *in vitro* (Bertsche et al., 2005; Born et al., 2006). The differences in PG synthesized in the presence of LpoA and LpoB *in vitro* may provide an explanation. This PG has significantly higher peptide cross-linkage than that observed in isolated sacculi, and contains high proportions of trimeric and tetrameric peptide structures never observed before *in vitro*. Although such highly crosslinked structures are rare in sacculi, they have been implicated in transient multi-layered PG present at growth sites where the newly synthesized glycan strands are connected to the sacculus, for example at the tip of the septum (Glauner and Höltje, 1990; Höltje, 1998). Thus, it is possible that LpoA/LpoB are required to control the attachment of newly synthesized PG strands to the existing sacculus *in vivo*, which is known to occur by the formation of cross-links between new and old peptides (Burman and Park, 1984; de Jonge et al., 1989; Glauner and Höltje, 1990). This idea is consistent with the demonstration by Paradis-Bleau et al. that depletion of both LpoA and LpoB *in vivo* leads to a decrease in peptide crosslinking.

### Why is PG synthesis regulated by OM-proteins?

Based on the PBP1B crystal structure, the small UB2H domain is less than ca. 60 Å away from the IM (Sung et al., 2009). As the distance between the IM and the PG layer is ca. 90 Å (Matias et al., 2003), the UB2H domain must be located in the space between the IM and the PG. Thus, the OM-bound LpoB must stretch *through* the pores in the PG net to interact with UB2H and activate PBP1B. It is intriguing to consider the possibility that Lpo-mediated activation of PBPs is responsive to the state of the pores in the PG net. PG pores act as a molecular sieve and are permeable to proteins of the appropriate size (Demchick and Koch, 1996; Vazquez-Laslop et al., 2001), and in growing *E. coli* cells, turgor stretches the PG significantly, which can expand up to 3-fold in surface area (Koch and Woeste, 1992; Yao et al., 1999). Likewise, PG might stretch and its pore size increase during rapid growth (rich media) as it happens during increased turgor (low osmolality; Cayley et al., 2000), and the converse might occur during slow growth (limited nutrients, stationary phase) and low turgor (high osmolality) thereby altering the efficiency with which Lpo proteins activate their cognate PBP through the pores. Such a homeostatic mechanism would continuously reset the rate of PG synthesis to overall cellular growth rate resulting in a PG layer with constant surface density and homogenous pore size as observed (Demchick and Koch, 1996). Other mechanisms are likely involved in the regulation of PG growth rate, thickness and surface density.

Alternatively, or in addition, OM localized Lpo proteins might recruit and/or control OM-anchored PG hydrolases (autolysins), which are responsible for the release of PG fragments during growth. The control of autolysins by Lpo proteins would ensure that the activity of these potentially dangerous enzymes is restricted to the sites of PG growth and is coupled to the activities of the synthases; such coupling of PG synthases and hydrolases has been proposed in a previous growth model (Höltje, 1998). Indeed, our preliminary data suggests that LpoB may recruit a PG hydrolase at septal sites. We are currently investigating the validity of our hypotheses.

### Redundancy and specialization of bifunctional PBPs; a dual role for PBP1B

PBP1A and PBP1B have partially redundant roles *in vivo* although they have different localization preferences. PBP1B has been suggested to be the major bifunctional PBP responsible for septal PG synthesis because of its septal localization and interactions with the essential cell division proteins PBP3 and FtsN (Bertsche et al., 2006; Müller et al., 2007), whereas PBP1A seems to be more active during cell elongation. LpoA and LpoB mirror the localization preferences of their cognate PBP, but localize independently of them. Septal localization of LpoB coincides with the presence of a mature divisome and depends on the presence of FtsZ, PBP3 and ongoing PG septal synthesis. Despite the localization preferences of the two complexes (Fig. 7B), there is some inherent flexibility in the system such that PBP1B-LpoB is able to perform sidewall PG synthesis in the absence of PBP1A-LpoA, and PBP1A-LpoA is able to take over septal PG synthesis in the absence of PBP1B-LpoB.

Although the two PG synthases generally substitute for each other, our results suggest that PBP1B is specifically required for cell division in certain conditions. When the Tol-Pal system is present, either PBP1B-LpoB or PBP1A-LpoA can mediate division. However, in the absence of Tol-Pal, under low salt conditions where the absence of Pal severely impacts cell division, PBP1B-LpoB is essential for viability and PBP1A-LpoA cannot substitute for its function. This suggests that PBP1B-LpoB compensates for Tol-Pal, most likely by contributing to OM constriction, and that PBP1A-LpoA is less proficient at compensation thus depending on the Tol-Pal system at all conditions. We do not exclude the possibility that additional systems exist that connect the OM to the IM and PG, localize at the septum, and facilitate OM constriction in *E. coli*. Recently, Tol-Pal was implicated in mediating OM constriction during cell division in *Caulobacter cresentus*, and was shown to be essential (Yeh et al., 2010). Interestingly, *C. cresentus* lacks LpoB and therefore would lack the PBP1B-LpoB backup system for OM constriction.

### A new evolutionary trait for PG synthesis in enteric bacteria

In contrast to the wide conservation of PBP1A and PBP1B, LpoA and LpoB are evolutionarily restricted. We have recently assessed growth profiles of the entire single gene deletion library of *E. coli* under a wide variety of conditions, and observed that for *E. coli*, the genes of unknown function that respond to many different conditions are generally restricted to the γ-proteobacteria (RJN, AT and CAG; manuscript submitted). In contrast, as expected, annotated genes that respond to many different conditions tend to be broadly distributed. An exciting explanation, consistent with the role of *lpoB* described in this work, is that such genes have been recently acquired to act as regulators of broadly conserved biological processes, adding an additional layer of control that helps the cell adjust to the specific needs of its niche.

### Concluding remarks

We have identified to our knowledge the first OM regulators of PG synthesis in bacteria. LpoA and LpoB are essential for the function of their cognate PBP *in vivo* and significantly stimulate the TPase activity of the cognate PBP *in vitro*. As neither LpoA and LpoB nor their cognate docking domains share sequence homology, this control mechanism must have evolved at least twice for γ-proteobacteria, which suggests that this a robust way to control PG synthesis. Other proteins, unrelated in sequence to LpoA/LpoB may perform similar functions in other bacterial phyla. PG synthases are a common antibiotic target; for example β-lactams target their TPase domains. Because LpoA or LpoB are evolutionarily confined, they could serve as more specific targets of a new generation of antibiotics that do not deplete the entire microbial flora of the patient, and/or could be administered together with β-lactams to increase the effectiveness of the latter and circumvent the activity of β-lactamases in the cell.

PG remodeling is emerging as a key developmental strategy for cells to adapt to environmental changes. Changes in the PG composition during stationary phase may trigger the disassembly of biofilms (Kolodkin-Gal et al., 2010; Lam et al., 2009), whereas tight regulation of PG hydrolases has been proposed to facilitate helical curvature and twist of *H. pylori* (Sycuro et al., 2010), spore morphogenesis in *B. subtilis* (Morlot et al., 2010) and septum formation in *E. coli* (Uehara et al., 2010). The common denominator of these reports and of our work is that bacteria have a complex network of PG synthases/hydrolases (and their regulators) to tailor PG architecture for optimal function in their niche. We have only begun to map these networks and understand their vast implications in bacterial lifestyle, but future research is likely to provide insights into how changes in PG architecture are integrated into developmental programs and the trafficking/assembly of large cell envelope components in the periplasm.

## Experimental Procedures

### Identification of PBP-interacting proteins

The chemical genetics screen is described in detail elsewhere (RJN, AT and CAG; manuscript submitted). In brief all single-gene knockouts of non-essential *E. coli* genes were subjected to a wide variety of conditions (including sub-lethal concentrations of drugs and environmental conditions) and their growth was quantitatively assessed after overnight growth at 37°C. The compendium of growth measurements across all conditions for a given gene was used to generate its phenotypic signature. Phenotypic signatures were then compared and used as a discovery tool for identifying genes that belong to the same biological process. The proteomics approach led to the identification of Lpo proteins is described in the Supplemental Information.

### Screen for genetic interactions

The 12×12 genetic interaction matrix was generated and analyzed using previously described protocols (Typas et al., 2008), except that mating and intermediate selection were done on M9 complete plates with 0.4% glycerol (with or without Kan), and 200 μl of donor cells at OD450=1 were plated as lawn for the mating step. For assessing genetic interactions between cognate lpo-mrc pairs, we first independently constructed the double mutants by P1 transduction. We then pinned wildtype, parental single mutant and double mutant cells in 384-format (n=96 colonies each) on LB agar plates containing different drugs that sensitized the parental single mutants. Raw colony size data were obtained by automated image analysis software, HT Colony Grid Analyzer (http://sourceforge.net/project/showfiles.php?group_id=163953). The expected growth of the double mutants was calculated as the product of the growth of the parental single mutants.

### In vitro PG synthesis assay

A published protocol (Bertsche et al., 2005) was used with minor changes. Different combinations of PBP1A (0.76 μM), PBP1B (0.74μM), LpoA (0.76 μM, LpoA^C^ (0.76 μM), LpoA^N^ (0.76 μM) and LpoB (0.69 μM) were pre-incubated for 15 min on ice in a total volume of 95 μl in 10 mM Hepes, 10 mM MgCl_2_, 150 mM NaCl pH 7.5. ^14^C-labelled lipid II (4.8 μM) was added, and the reaction proceeded for 1 h at 30°C or 37°C. Muropeptides were prepared and analyzed by HPLC as described (Bertsche et al., 2005). Attachment of newly synthesised PG to sacculi was assayed as described in (Born et al., 2006).

### Other experimental procedures

All other experimental procedures applied in this study are based on previously published methodology and any modifications used are described in detail in the Supplementary Information. Growth conditions, strains and plasmids used in this study can be also found in the Supplementary Material.

## Supplementary Material

01

02
