# Supplementary material for: Regulation of peptidoglycan synthesis by outer membrane proteins
Source: Cell. Author manuscript; Available in PMC 2011 Dec 23. (PMC3060616; doi:10.1016/j.cell.2010.11.038)
Supplement: 01 [file NIHMS263303-supplement-01.pdf]

i. <u>Supplemental Data</u>

Figure Legends:

Figure S1. Genetic interactions between *lpo* and *mrc* genes (see also Fig. 1). A-B. *lpoA*$^-$ is synthetically lethal with both *mrcB*$^-$ and *lpoB*$^-$. Results from pseudo-Hfr *lpoA*::*cat* crossed with 12 Kan^R recipients arrayed in 1536 format (boxes of 4x32=128 replicas) on agar plates are shown in (**A**) and quantified in (**B**). Recipients are indicated above the double mutant plate shown here. The self mating control (*lpoA*::*cat* x *lpoA*::*kan*; red), demonstrates the low false-positive rate; the white box is a sterility control. *lpoA*$^-$ is synthetically lethal with *mrcB*$^-$ and *lpoB*$^-$, but not with other genes; the interaction with *yraI*$^-$ is due to linkage of the two genes, which reduces crossover frequency and recover of double mutants (*lpoA* and *yraI* are 4.5 kb apart). Double mutants of *lpoA* with members of the Tol-Pal system exhibit mucoid colonies, which result into bigger colony sizes but do not reflect a real increase in fitness (fitness of these double mutants was assessed in liquid cultures and the *lpoA*$^-$-*pal*$^-$ and *lpoA*$^-$-*tolQ*$^-$ genetic interactions were marginally negative; data not shown). Error bars depict standard deviations (n=128). **C-F.** *lpoB*$^-$ and *lpoA*$^-$ show epistatic genetic interactions with *mrcB*$^-$ (C-E) and *mrcA*$^-$ (F) respectively. The wildtype (position 1), single *lpo* mutant (position 2), single *mrc* mutant (position 3) and double mutant strain (position 4) are arrayed in 384-format (n=96 colonies each) on LB agar plates containing Carbenicillin 1 µg/ml (**C**), Mecillinam 0.12 µg/ml (**D&F**) or Cefsulodin 6 µg/ml (**E**). Panels C-E contain the *lpoB-mrcB* pair and panel F the *lpoA-mrcA* pair. Quantifications of the 4 plates are shown in Fig. 1D-E.

Figure S2. LpoA and LpoB are OM localized and interact with PG sacculi.
A. *E. coli* IM and OM vesicles were separated by 35-60% sucrose density gradient centrifugation (upper panel). Proteins in the isolated membrane fractions were separated by SDS-PAGE and blotted on nitrocellulose. Lpo proteins as well as control proteins with known localization (MltA, OM; MurG, IM) were immunodetected with specific antisera. Both Lpo proteins localized to OM vesicles. **B.** N-terminal amino acid

sequence of LpoA (SwissProt accession No. P45464) and LpoB (SwissProt accession No. P0AB38). The hydrophobic part of the signal peptide is highlighted. The amino acids in the lipoprotein signature sequence are in bold and underlined, with the cysteine residue for lipid modification shown in red. **C.** Purified Lpo proteins were incubated with or without sacculi followed by sedimentation of the sacculi by ultracentrifugation. Sacculi were washed and sedimented again before proteins were separated by SDS-PAGE and visualized by Coomassie-staining. A fraction of each Lpo protein was pulled-down with PG sacculi. S, supernatant after the first centrifugation; W, supernatant after the washing step; P, resuspended pellet; M, protein size markers. Fig. 2 shows the interaction of the Lpo proteins with their cognate PBP.

Figure S3. Lpo proteins stimulate the TPase activity of their cognate PBP in vitro. A. Flow diagram of the in vitro PG synthesis assay. The GTase and TPase domains of the bi-functional class A PBP polymerize the radioactively labeled lipid II substrate. The product is boiled to remove the lipid anchor and hydolysed by the muramidase cellosyl to produce the mono-, di-, tri- and tetrameric muropeptides. The muropeptides are reduced by $NaBH_4$ and then separated by HPLC connected to a radioactivity flow-through detector. G, GlcNAc; M, MurNAc; M(r), reduced MurNAc; green bar, peptide; *P*, phosphate group; upr, undecaprenyl residue. **B.** HPLC profiles of the muropeptides generated from in vitro synthesized PG. The PBP and Lpo proteins present in the reaction are indicated on the right side. Numbers of the peaks correspond to the structures shown in (**C**). Note that reaction conditions for PBP1A differed from previously published ones (Born et al., 2006) resulting in higher TPase activity of PBP1A alone. At this improved condition, PBP1A has also DD-carboxypeptidase activity leading to the formation of tetrapeptides (MB and WV, manuscript in preparation). **C.** Proposed structures of muropeptides synthesized *in vitro* by PBP1A and PBP1B. Fig. 3F contains a summary of the quantified muropeptide peaks shown in Fig. S3B.

Figure S4. LpoA stimulates the attachment of newly synthesized PG to sacculi in vitro. A. Schematic representation of the attachment reaction in which radioactively labeled lipid II substrate is polymerized by the GTase and TPase activities of the

bifunctional PBP1A. In the presence of non-labeled PG sacculi part of the newly synthesized, radiolabeled material becomes covalently attached to sacculi. After centrifugation, the radioactivity in supernatant and pellet is quantified to determine attachment efficiency. Abbreviations are the same as in Fig. S3. **B.** Attachment efficiency of PBP1A alone and PBP1A with the different LpoA variants. The values are mean +/- standard deviation of three independent experiments. The p-values determined by analysis of variance (ANOVA) are given above the columns. The presence of LpoA, but not of $LpoA^N$ or $LpoA^C$, increased the percentage of newly made PG that becomes attached to sacculi. Both Lpo proteins stimulate the TPase activity of their cognate PBP in the absence of sacculi (Fig. 3F).

Figure S5. Immunolocalization protocol does not affect the cell membrane; LpoA and LpoB localize late in the cell cycle at midcell. A. The membranes of non-immunolabeled and immunolabeled fixed cells were stained with BODIPY 558/568. Fluorescence intensity profiles were collected from wildtype cells in TY at 28°C. Quantitative analysis of ~500 cells reveals the membrane staining of both cell types (immunolabeled-solid lines; non-immunolabeled-dashed lines) is practically identical, suggesting that our immunolocalization protocol does not perturb the cell membranes. **B-C.** Fluorescence profiles were collected from wildtype cells grown in GB1 to steady state at 28°C. The profiles were sorted by cell length and averaged in batches of ~10% of the cells going from small to long cells. For each fraction of the cells, the age of the cell was calculated. Each profile shown above corresponds to the average of >500 cells, which have on average, the ages that correspond to the indicated percentage of the cell cycle. The profiles have been artificially drawn with increasing background, to be able to visualize each profile (in reality the profiles overlap). FtsZ localizes after 39% of the cell cycle and the late localizing proteins localize after 59% of the cell cycle in this type of cell (LMC500 derivatives). Profiles of LpoA cells are indicative of absence of label in the poles and of a slight mid-cell localization after 74.5% of the cell cycle (**B**), which presumably corresponds to a doubling of the membrane due to septum formation. Profiles of LpoB cells exhibit pronounced mid-cell localization after 62.5% of the cell

cycle (**C**). LpoB, and to a smaller degree LpoA, exhibit midcell localization that is independent of the presence of PBP1B or PBP1A (Fig. 4).

Figure S6. Localization of LpoA and LpoB in FtsZ (ts) cells (LMC509) and PBP3 (ts) cells (LMC510) grown in minimal glucose medium, and in wildtype (LMC500) cells grown in TY medium upon inhibition of PBP3 with Aztreonam. A & D. LMC509 at the permissive temperature of 28°C. **B & E.** LMC509 shifted for two doubling times at 42°C. **G & J.** LMC510 at the permissive temperature of 28°C. **H & K.** LMC510 shifted for two doubling times at 42°C. **M & N.** LMC500 at 28°C before and 45 min after treatment with Aztreonam. **A, B, G**, and **H** were incubated with antibodies against LpoA; **D, E, J, K, M** and **N** were incubated with antibodies against LpoB. Each panel consists of a phase contrast and a fluorescence image. The scale bar represents 5 μm. Panels **C** (LMC509 anti LpoA), **F** (LMC509 anti LpoB), **I** (LMC510 anti LpoA) and **L** (LMC510 anti LpoB) are the fluorescence profiles of >1000 cells of the samples shown in the microscopy images. The solid lines are from the 28°C cells and the dashed lines and dotted lines are from the cells grown for 1 and 2 mass doublings at 42°C, respectively. Panel **O** (LMC500 anti LpoB) also contains the fluorescence profiles of >1000 cells of the samples shown in the microscopy images in **M** and **N**. The black line is from cells before Aztreonam addition and the grey line is from cells 45 min after Aztreonam addition. LpoB loses its septal localization upon depletion of FtsZ or FtsI, and 45 min after treatment with Aztreonam, but not in the absence of any of PBP1A or PBP1B (Fig. 4). Note that the fluorescence signal in minimal medium grown cells (**A-L**) is weaker compared to TY grown cells (**M-O**) due to either smaller cell sizes and thus lower number of Lpo molecules or due to a specific nutritional or osmotic effect.

Figure S7. Cells dependent on PBP1B can tolerate an IM version of LpoB only under no/low salt conditions. OD_{578} of various strains measured following overnight growth in LB with different amounts of salt. Error bars depict standard deviation (n>6). *lpoB*$_{IM}$*mrcA*$^-$ with IM-localized LpoB$_{IM}$ lacking PBP1A is viable at LB without salt, but lyses at low/moderate salt concentrations and is essentially non-viable at higher salt

concentrations. At the same conditions that $LpoB_{IM}$ is functional (no/low salt conditions), it cannot substitute for the loss of Pal and support OM invagination (Fig. 5).

Table S1. List of strains used in this study (see also Supplemental Experimental Procedures)

Strain	Genotype	Notes/References
BL21 (DE3)	Expression strain F- *ompT*, *dcm*, *hsdS* (rB- mB-) *gal* λ(DE3)	Novagen
TB28	MG1655 *lacIZYA*	(Taschner et al., 1988)
MC4100	F^- *araD139 Δ(argF-lac)U169 deoC1 flbB5301 ptsF25 rbsR relA1 rpsL150*	(Casadaban, 1976)
MC1061	F^- Δ(*ara-leu*)7697 [*araD*139]$_{B/r}$ *Δ(codB-lacI)*3 *galK*16 *galE*15 $λ^-$ e14$^-$ *mcrA*0 *relA*1 *rpsL150(strR) spoT1 mcrB1 hsdR2* (r^- m^+)	(Casadaban and Cohen, 1980)
LMC500	MC4100 *lysA1*	(Taschner et al., 1988)
LMC509	LMC500 *ftsZ84*(Ts)	(Taschner et al., 1988)
LMC510	LMC500 *ftsI2158* (Ts)	(Taschner et al., 1988)
BW25113	*rrnB3 ΔlacZ4787 hsdR514 Δ(araBAD)567 Δ(rhaBAD)568 rph-1*	backbone of KEIO collection; (Baba et al., 2006; Yamamoto et al., 2009)
BW38029	*rrnB3 ϕ (lacZp4105(UV5)-lacY)638 ΔlacZhsdR514 Δ(araBAD)567 rph*$^+$	backbone of ASKA collection, unpublished; Mori H & colleagues
Keio single mutants	*ycfM::Kan, yraM::kan, mrcA::kan, mrcB::kan, pal::kan, tolA::kan, tolQ::kan, tolR::kan, tolA::kan, tatB::kan, envC::kan, ftsP::kan*	(Baba et al., 2006; Yamamoto et al., 2009)
Aska single mutants	*pal::cat, mrcA::cat, mrcB::cat, yraM::cat, ycfM::cat*	unpublished; Mori H & colleagues
CAG60337	BW25113 *yraM::kan mrcA::cat*	this work
CAG60338	BW25113 *ycfM::kan mrcB::cat*	this work
CAG60339	BW25113 *ycfM::kan mrcA::cat* pBAD30 *lpoB*	this work
CAG60340	BW25113 *yraM::kan mrcB::cat* pBAD30 *lpoA*	this work
CAG60345	BW38029 *yraM::cat mrcB::kan* pBAD30 *lpoA*	this work
CAG60361	BW25113 *ycfM::kan mrcA::cat* pBAD30 *lpoB*IM	this work
CGA60366	BW25113 *mrcB ΔUB2H*	this work; original chromosomal copy missing amino acids E114-Q191
CAG60367	BW25113 *ycfM::kan pal::cat* pBAD30 *lpoB*	this work
CAG60368	BW25113 *ycfM::kan pal::cat* pBAD30 *lpoB* IM	this work
CAG60377	BW25113 *ycfM IM*	this work;original chromosomal copy with 2 amino acid exchanges in its lipo-sorting signal (D+2, E+3)
CAG60379	BW25113 *ycfM IM mrcA::kan*	this work
CAG60380	BW25113 *ycfM IM mrcB::kan*	this work
CAG60381	BW25113 *ycfM IM yraM::kan*	this work
CAG60382	BW25113 *ycfM IM pal::cat*	this work
CAG60396	BW25113 *ycfM::kan pal::cat*	this work

Table S2. List of plasmids used in this study (see also Supplemental Experimental Procedures)

Plasmid	Notes
pBAD30LpoA	*lpoA* cloned between EcoRI and SphI: used in depletion experiments; Fig. 3
pBAD30LpoB	*lpoB* cloned between EcoRI and SphI: used in depletion experiments; Fig. 3
pBAD30LpoB IM	IM LpoB variant (D+2, E+3 lipoprotein sorting signal), gene cloned between EcoRI and SphI
pQE30-ssODD (pssODD in the ms)	ODD domain of PBP1A fused to an N-terminal hydrophobic variant of the LamB signal sequence; fusion gene cloned between EcoRI and HindIII; expressed fusion protein: MMITLRKLPLAVIVLLVVMSAQAMAV-MrcA(G^{315}-V^{422}): used in Fig. 6 as pssODD
pBAD33LpoA	*lpoA* cloned between EcoRI and SphI; used in Fig. 6 as pLpoA
pBAD33LpoB	*lpoB* cloned between EcoRI and SphI
pET28LpoB	*lpoB* region encoding the soluble part of LpoB cloned between NdeI and HindIII; expressed fusion protein: MGSSH$_6$SSGLVPRGSHM-LpoB(V^{21}-Q^{231})
pET28LpoA	gene region encoding the soluble fraction of LpoA cloned between NdeI and HindIII; expressed fusion protein: MGSSH$_6$SSGLVPRGSQAYM-LpoA(G^{28}-S^{678})
pET28LpoA^N	gene region encoding the soluble N-terminal fraction of LpoA cloned between NdeI and HindIII; expressed fusion protein: MGSSH$_6$SSGLVPRGSHM-LpoA(G^{28}-T^{256})
pET28LpoA^C	gene region encoding the soluble C-terminal fraction of LpoA cloned between NdeI and HindIII; expressed fusion protein: MGSSH$_6$SSGLVPRGSHSAYM-LpoA(N^{257}-S^{678})

ii. Supplemental Experimental Procedures

Growth conditions, bacterial strains and plasmids

Cells were grown in LB Lennox medium at 37°C unless mentioned otherwise, and where appropriate, antibiotics were used at 20 (Cam), 30 (Kan) or 100 (Amp) µg/ml. For strains containing derivatives of the overexpression vectors pBAD and pQE, cells were grown in the presence of 10 mM arabinose and 0.5 mM IPTG, respectively, to achieve full induction. All strains used in in-vivo experiments in this study (Table S1) are derivatives of the closely related BW25113 and BW38029, apart from the ts and *lpoA*$^-$ strains used for localization experiments, which are MC4100 and TB28 derivatives, respectively. For most in-vitro experiments, unless mentioned otherwise, MC1061 was used as a host strain. All deletion mutants stem either from the KEIO (kan^R; (Baba et al., 2006; Yamamoto et al., 2009) or ASKA (cat^R-unpublished library from H. Mori's lab) collections. When necessary, deletion strains were transduced to other backgrounds

using P1 phage, whereas Hfr donors were made using our “double male” strain (Typas et al., 2008). Allele specific exchanges in LpoB and deletions in PBP1B are scarless mutations in the original chromosomal locus. The scarless mutations were made by first introducing a *sacB*::*kan* cassette in the targeted locus in the genome of a strain carrying pKD46 (Datsenko and Wanner, 2000), which was then replaced by electroporating the new DNA fragment that encompassed the desired point mutations/deletions and selecting for Kan^S clones in 5% sucrose. The λ_{RED} plasmid (pKD46) was cured and clones were verified by PCR and sequencing. Plasmids used in this study are shown in Table S2. Primers used for the construction of new plasmids and strains are available upon request.

Proteomics-based identification of PBP-interacting proteins

Cell membranes from *E. coli* strain MC1061 were extracted with 10 mM Tris pH 7.2, 10 mM $MgCl_2$, 1 mM NaCl, 1 mM EGTA, 2% Triton X-100, 200 µM PMSF, pepstatin/antipain/leupeptin/aprotinin cocktail -1 µg/ml each- (extraction buffer), and the extract was cleared by ultracentrifugation. The cleared extract was dialysed against extraction buffer with 100 mM or 500 mM NaCl. Purified PBP1A, PBP1B, PBP2 or PBP3 (30 µg of each) were coupled to 75 µl of Affigel-10 gel beads (Sigma). Coupling was tested by SDS-PAGE of the supernatant samples before and after coupling. Control beads contained no protein. For each protein and the control, the beads were divided into three equal portions, for incubation with membrane extract with 100 mM, 500 mM NaCl, or membrane extraction buffer (100 mM NaCl) as a control. For each affinity chromatography against the Affigel-10 coupled PBPs, 100 µl of the dialysed extract containing the extracted proteins from 670 µg MC1061 membranes was incubated for 20 h at 4°C under gentle agitation. The beads were transferred into new tubes and extensively washed with 10 mM Tris pH 8.0, 10 mM $MgCl_2$, 100 mM NaCl, and 0.05% Triton X-100. Then, the beads were extracted with 100 µl of 10mM Tris pH 8.0, 500 mM NaCl, and 0.2% N-Lauroylsarcosine (sarcosyl). Proteins in the sarcosyl extract were precipitated adding 3 volumes of ethanol, followed by overnight incubation at -20°C. Proteins were pelleted by centrifugation and air-dried. Proteins were identified

by trypsin digestion and peptide mass fingerprinting as previously described (von Rechenberg et al., 2005).

Protein purification of Lpo and PBP proteins

BL21 (DE3) cells were used for overexpressing all proteins. Recombinant PBP1A and PBP1B were purified as described previously (Bertsche et al., 2005; Born et al., 2006). For Lpo proteins 3 l of LB auto-induction medium (10 g/l tryptone, 5 g/l yeast extract, 1 mM $MgSO_4$, 10 g/l NaCl, 0.5% glycerol, 0.05% glucose, 0.02% lactose, pH 7.2 – 7.5) containing 50 µg/ml Kan was inoculated with 50 ml of an overnight culture carrying the appropriate plasmid (pET28 derivatives). Cells were grown for 18 h at 30°C and then harvested by centrifugation (8000×*g*, 10 min, 4°C). The cell pellet was resuspended in 40 ml of buffer I (25 mM Tris/HCl, 10 mM $MgCl_2$, 200 mM NaCl, pH 7.5). A small amount of DNase was added to the cells before they were disrupted by sonication. Cell membranes were pelleted by ultracentrifugation (80000×*g*, 45 min, 4°C). The supernatant was supplemented with 20 mM imidazole and incubated with 3 ml Ni-NTA superflow beads (Qiagen) for 16 h at 4°C. The beads were allowed to settle in a gravity column and were washed with 100 ml wash buffer (25 mM Tris/HCl, 10 mM $MgCl_2$, 200 mM NaCl, 10% glycerol, 20 mM imidazole, pH 7.5). Retained proteins were eluted with 10 ml elution buffer (25 mM Tris/HCl, 10 mM $MgCl_2$, 200 mM NaCl, 10% glycerol, 400 mM imidazole, pH 7.5). Proteins were dialysed against dialysis buffer (25 mM Tris/HCl, 10 mM $MgCl_2$, 250 mM NaCl, 10% glycerol, pH 7.5). For a second purification step LpoA and LpoB were dialysed against LpoA buffer A (20 mM Tris/HCl, pH 8.0) and LpoB buffer A respectively (10 mM Tris/maleate, pH 5.2). The proteins were applied to a 5 ml High Trap Mono Q HP column (GE) at a flow rate of 0.5 ml/min using an ÄKTA prime FPLC. LpoA & LpoB were eluted in a linear gradient of 45 ml from 100% LpoA/LpoB buffer A to 100% LpoA buffer B (20 mM Tris/HCl, 500 mM NaCl, pH 8.0) or 100% LpoB buffer B (25 mM Tris/maleate, 1 M NaCl, pH 5.2) at a flow rate of 0.5 ml/min. Fractions containing LpoA and LpoB were dialysed against dialysis buffer (25 mM Tris/HCl, 10 mM $MgCl_2$, 250 mM NaCl, 10% glycerol, pH 7.5 for LpoA and pH 8.0 for LpoB).

Generation and purification of Lpo antisera

Serum against LpoA and LpoB was produced from rabbits by Eurogentec (Herstal, Belgium), using LpoA or LpoB protein for immunisation. Lpo antibodies were affinity-purified as described (Bertsche et al., 2006).

Preparation of membrane fraction for affinity chromatography

This protocol was adapted from a published method (Vollmer et al., 1999). Membranes were isolated from 2 l of *E. coli* MC1061 grown at 37°C to an OD (578 nm) of 0.5 to 0.6. Cells were harvested (4000×g, 10 min, 4°C), resuspended in 10 ml of MF buffer I (10 mM Tris/maleate, 10 mM $MgCl_2$, pH 6.8) and disrupted by sonication. Membranes were sedimented by ultracentrifugation (80000×*g*, 45 min, 4°C). The pellet was resuspended in 10 ml of MF buffer II (10 mM Tris-maleate, 10 mM $MgCl_2$, 1 M NaCl, 2% Triton X-100, pH 6.8) to extract all membrane proteins by stirring over-night at 4°C. The supernatant obtained after another ultracentrifugation step (80000×*g*, 45 min, 4°C), was diluted by the addition of 10 ml of MF dialysis buffer I (10 mM Tris/maleate, 10 mM $MgCl_2$, 400 mM NaCl, pH 6.8) and dialysed against 3 l of the same buffer. The obtained membrane fraction was used directly for affinity chromatography.

Affinity chromatography with immobilized PBPs and Lpo proteins

This protocol was adapted from a previously published method (Vollmer et al., 1999). Briefly, 10 mg of protein (PBP1A, PBP1B, LpoA, or LpoB) were coupled to 0.9 g of CNBr-activated sepharose by gentle agitation over-night at 6°C. The remaining coupling sites were blocked with AC blocking buffer (200 mM Tris/HCl, 10 mM $MgCl_2$ 500 mM NaCl, 10% glycerol and 0.25% Triton X-100, pH 7.4) by incubation with gentle agitation over-night at 6°C. The beads were washed alternating with AC blocking buffer and AC acetate buffer (100 mM sodium acetate, 10 mM $MgCl_2$, 500 mM NaCl, 10% glycerol and 0.25% Triton X-100, pH 4.8), and finally resuspended in AC buffer I (10 mM Tris/maleate, 10 mM $MgCl_2$, 50 mM NaCl, 1% Triton X-100, pH 6.8). As control (Tris-Sepharose) one batch of activated Sepharose was treated identically, except that no protein was added. Affinity chromatography was performed at 6°C. The membrane fraction containing 400 mM NaCl was incubated with gentle agitation overnight. The

column was washed with 50 ml of AC wash buffer (10 mM Tris/maleate, 10 mM $MgCl_2$, 400 mM NaCl and 0.05% Triton X-100, pH 6.8). Retained proteins were eluted with 20 ml of AC elution buffer (10 mM Tris/maleate, 10 mM $MgCl_2$, 2 M NaCl, 0.05% Triton X-100, pH 6.8). Proteins present in the fractions were separated by SDS-PAGE, blotted and immunodetected as described (Bertsche et al., 2006).

In vivo cross-linking and co-immunoprecipitation

A slightly modified version of the published procedure (Müller et al., 2007) was used. *E. coli* MC1061 cells were grown in 150 ml of LB medium at 37°C to an OD of 0.6. Cells were harvested by centrifugation (4000×*g*, 15 min, 4°C) and resuspended in 6 ml cold CL buffer (50 mM NaH_2PO_4, 20% sucrose, pH 7.4). Freshly prepared DTSSP solution (20 mg/ml in H_2O) was added and cells were incubated for 1 h. Cross-linked cells were harvested by centrifugation (4000×*g*, 15 min, RT) and resuspended in 8 ml of CL buffer II (100 mM Tris/HCl, 10 mM $MgCl_2$, 1 M NaCl, pH 7.5). Then 1 µg/ml of protease inhibitor cocktail (Sigma) and a small amount of DNase were added. The cells were disrupted by sonication and membranes were sedimented by ultracentrifugation (90000×*g*, 60 min, 4°C) and resuspended in CL buffer III (25 mM Tris/HCl, 10 mM $MgCl_2$, 1 M NaCl, 20% glycerol, 2% Triton X-100, pH 7.5). Membrane extraction was done overnight at 6°C under mild stirring. After another centrifugation step (90000×*g*, 60 min, 4°C) the supernatant was taken and diluted with CL buffer IV (75 mM Tris/HCl, 10 mM $MgCl_2$, 1 M NaCl, pH 7.5). 5-15 µg of the specific antibodies were added and the sample was incubated for 5 h at 4°C. As a control, half of the sample was incubated without antibody. Protein G-coupled agarose (40 µl suspension) was added to the membrane fraction and the sample was incubated overnight at 4°C. The beads were centrifuged and the supernatant sample was collected. The beads were then washed with 10 ml of CL wash buffer (60 ml of CL buffer III + 30 ml of CL buffer IV) and boiled for 8 min in 50 µl of sample buffer for SDS-PAGE. The supernatant was collected (protein G sample) and analysed by SDS-PAGE followed by Western Blot and immunodetection.

Cell microscopy

Cells were grown until an O.D. of 0.5 to 0.8 was reached. 500 µl of the cells were mixed with 40 µl of 0.5 M Na_2HP0_4 adjusted with H_3PO_4 to a pH of 7.4, 100 µl 16% of para-formaldehyde and 0.4 µl of 25% glutaraldehyde. The suspension was incubated 15 min at RT and 30 min on ice. The cells were centrifuged (14000x*g*) and the pellet washed twice with 1 ml of PBS. After the second wash step the cells were resuspended with PBS to an OD of 1.0 and stored at 4°C. Fixed cells were examined on an inverted epifluorescence microscope (Zeiss Axiovert 200M) fitted with a Plan-Neofluar objective (Zeiss 100×/1.30 Oil Ph3), a 300W xenon arc-lamp transmitted through a liquid light guide (Sutter Instruments), and a Sony CoolSnap HQ cooled CCD camera (Roper Scientific).

Immunolabelling of LpoA and LpoB

First, we present a detailed description of the method and then we shortly discuss our rationale for electing immunolabelling to detect localization of the Lpos, and additional controls we have performed.

E. coli cells were grown at 28°C in rich medium or in minimal glucose medium as described before (Aarsman et al., 2005). Immunolocalization of LpoA and LpoB in various *E. coli* strains was performed as described previously (den Blaauwen et al., 2001). Fixed and permeabilized cells were incubated for 1 hour at 37°C with affinity purified polyclonal antibodies directed against LpoA or LpoB diluted 1:1000 in blocking buffer. Donkey anti-rabbit conjugated to CY3 (Jackson Immunochemistry, USA) diluted 1:300 in blocking buffer was used as a secondary antibody and incubated for 30 minutes at 37°C. For immunolocalization, cells were immobilized on 1% agarose in water slabs coated object glasses as described (Koppelman et al., 2004) and photographed with a Coolsnap *fx* (Photometrics) CCD camera mounted on an Olympus BX-60 fluorescence microscope through a 100x/N.A. 1.35 oil objective. Images were taken using the program 'Object-Image2.19 by Norbert Vischer (University of Amsterdam, http://simon.bio.uva.nl/object-image.html), which is based on NIH Image-J by Wayne Rasband. The fluorescence profiles of the cells were analyzed using the public domain program 'Object-J 0.98a' by Norbert Vischer (University of Amsterdam,

http://simon.bio.uva.nl/object-J.html), which is based on ImageJ by Wayne Rasband as described (Potluri et al., 2010).

We visualized the Lpo proteins by immunolocalization rather by fluorescent fusion proteins because we wished to examine the location of native levels of untagged proteins thereby reducing artefacts from ectopic overexpression of a fusion protein. Although each method has limitations, we note that: a) in the largest-scale localization study in bacteria to date, the Gitai lab (Werner et al., 2009) found only 58/289 proteins (20%) to retain their localization both with an N- or C-terminal fluorescent fusion (289/2786 proteins tested exhibited specific localization; 58/289 showed the same localization for both N- and C-terminus fusions); b) proteins that localize at midcell (as we propose for LpoB), are often recruited by other proteins or by ongoing septal synthesis; ectopic/higher expression of LpoB may artificially change its localization because its recruiting protein or the septal signal is limiting.

For our experiments, the cells were fixed and subsequently the OM and PG were made permeable to antibodies by Triton X-100 and lysozyme, respectively. Many studies have documented that this procedure does not alter the localization pattern of proteins, nor does it cause plasmolysis of the cells (for examples see Martin et al., 2004; Morlot et al., 2003; Scheffers et al., 2004). We document below our additional controls to assess whether our protocol perturbed the native state of the cell.

(1) The average length (aL) and the average diameter (aD) of cells directly after the instantaneous fixation procedure and after the complete immunolocalization procedure were practically identical, demonstrating that the immunolocalization procedure does not affect shape.

Fixation only: aL = 4.14 ± 1.07 μm and aD = 1.00 ± 0.06 μm, n = 497 cells

Immunolabeled: aL = 4.05 ± 0.94 μm and aD = 1.09 ± 0.07 μm, n = 582

(2) Confocal microscopy demonstrated that immunolabeled cells had a perfect cylindrical shape (data not shown).

(3) Quantitative analysis of the fluorescence intensity profiles of fixed and BODIPY 558/568 membrane-stained cells with or without immunolabeling were practically identical (Fig. S5A).
(4) Septal localization of LpoB was also observed even when lysozyme was omitted from our protocol (our unpublished data).
Taken together, these additional controls make it highly unlikely that the immunolabeling protocol itself causes LpoB, and to a lower degree, LpoA to localize at midcell of dividing cells. Instead, our quantitative methodology based on native amounts of unlabeled proteins might enable us to capture localization preferences of these proteins.

Assay for binding of protein to PG

Binding of proteins to PG was assayed as described (Ursinus et al., 2004) with minor modifications. A sample of 100 µl of a 1 mg/ml PG suspension of *E. coli* MC1061 was pelleted by centrifugation (13000×*g*, 15 min, 4°C). The supernatant was discarded and the pellet resuspended in binding buffer (10 mM Tris/maleate, 10 mM $MgCl_2$, 50 mM NaCl, pH 6.8). Thereafter 10 µg of protein were added to a total volume of 100 µl. A control sample was prepared without PG. Samples were incubated for 30 min on ice and then pelleted by centrifugation (13000×*g*, 15 min, 4°C). The supernatant was collected and the PG pellet was resuspended in 200 µl of ice-cold binding buffer, followed by an additional centrifugation step. The supernatant (wash fraction) was collected and the pellet was resuspended in 2% SDS, followed by 1 h stirring. The supernatant of the binding step, the wash and SDS suspension were analysed by SDS-PAGE.

Separation of IM and OM vesicles

The protocol was adopted from a published method (Ehlert et al., 1995). Membranes were isolated from 750 ml of *E. coli* MC1061 grown at 37°C to an OD (578 nm) of 0.6. Cells were harvested at (5000×*g*, 10 min, 4°C) and resuspended in 10 ml of 50 mM sodium phosphate buffer, pH 7.0. Protein inhibitor cocktail (1:1000) and a small amount of DNase were added before cells were mechanically disrupted in a French press at 18000 psi. Unbroken cells were removed by centrifugation (500×*g*, 20 min, 4°C).

Membranes were harvested by centrifugation (90000×*g*, 45 min, 4°C) and resuspended in 300 µl of ice-cold 50 mM Na_3PO_4 buffer, pH 7.0. The membranes were applied to a sucrose gradient of 2 ml 70%, 1 ml 65%, 1 ml 60%, 1 ml 55%, 1 ml 50%, 1 ml 45%, 1 ml 40%, 1 ml 35% and 1 ml 30% sucrose in 50 mM Na_3PO_4 buffer, pH 7.0, and then centrifuged (150000×*g*, 20 h, 4°C). OM and IM vesicles were collected and stored at -20°C for further analysis.

Other methods

PG sacculi were prepared as described (Glauner et al., 1988); [^{14}C]Glc*N*Ac-labelled lipid II was prepared as described (Breukink et al., 2003).

iii. Supplemental References

Aarsman, M.E., Piette, A., Fraipont, C., Vinkenvleugel, T.M., Nguyen-Disteche, M., and den Blaauwen, T. (2005). Maturation of the *Escherichia coli* divisome occurs in two steps. Mol Microbiol *55*, 1631-1645.

Baba, T., Ara, T., Hasegawa, M., Takai, Y., Okumura, Y., Baba, M., Datsenko, K.A., Tomita, M., Wanner, B.L., and Mori, H. (2006). Construction of *Escherichia coli* K-12 in-frame, single-gene knockout mutants: the Keio collection. Mol Syst Biol *2*, 2006 0008.

Bertsche, U., Breukink, E., Kast, T., and Vollmer, W. (2005). In vitro murein peptidoglycan synthesis by dimers of the bifunctional transglycosylase-transpeptidase PBP1B from *Escherichia coli*. J Biol Chem *280*, 38096-38101.

Bertsche, U., Kast, T., Wolf, B., Fraipont, C., Aarsman, M.E., Kannenberg, K., von Rechenberg, M., Nguyen-Disteche, M., den Blaauwen, T., Höltje, J.-V.*, et al.* (2006). Interaction between two murein (peptidoglycan) synthases, PBP3 and PBP1B, in *Escherichia coli*. Mol Microbiol *61*, 675-690.

Born, P., Breukink, E., and Vollmer, W. (2006). In vitro synthesis of cross-linked murein and its attachment to sacculi by PBP1A from *Escherichia coli*. J Biol Chem *281*, 26985-26993.

Breukink, E., van Heusden, H.E., Vollmerhaus, P.J., Swiezewska, E., Brunner, L., Walker, S., Heck, A.J., and de Kruijff, B. (2003). Lipid II is an intrinsic component of the pore induced by nisin in bacterial membranes. J Biol Chem *278*, 19898-19903.

Casadaban, M.J. (1976). Transposition and fusion of the *lac* genes to selected promoters in *Escherichia coli* using bacteriophage lambda and Mu. J Mol Biol *104*, 541–555.

Casadaban, M.J., and Cohen, S.N. (1980). Analysis of gene control signals by DNA fusion and cloning in *Escherichia coli*. J Mol Biol *138*, 179-207.

Datsenko, K.A., and Wanner, B.L. (2000). One-step inactivation of chromosomal genes in *Escherichia coli* K-12 using PCR products. Proc Natl Acad Sci U S A *97*, 6640-6645.

den Blaauwen, T., Lindqvist, A., Löwe, J., and Nanninga, N. (2001). Distribution of the *Escherichia coli* structural maintenance of chromosomes (SMC)-like protein MukB in the cell. Mol Microbiol *42*, 1179-1188.

Ehlert, K., Höltje, J.-V., and Templin, M.F. (1995). Cloning and expression of a murein hydrolase lipoprotein from *Escherichia coli*. Mol Microbiol *16*, 761-768.

Glauner, B., Höltje, J.-V., and Schwarz, U. (1988). The composition of the murein of *Escherichia coli*. J Biol Chem *263*, 10088-10095.

Koppelman, C.M., Aarsman, M.E., Postmus, J., Pas, E., Muijsers, A.O., Scheffers, D.J., Nanninga, N., and den Blaauwen, T. (2004). R174 of Escherichia coli FtsZ is involved in membrane interaction and protofilament bundling, and is essential for cell division. Mol Microbiol *51*, 645-657.

Martin, M.E., Trimble, M.J., and Brun, Y.V. (2004). Cell cycle-dependent abundance, stability and localization of FtsA and FtsQ in *Caulobacter crescentus*. Mol Microbiol *54*, 60-74.

Morlot, C., Zapun, A., Dideberg, O., and Vernet, T. (2003). Growth and division of *Streptococcus pneumoniae*: localization of the high molecular weight penicillin-binding proteins during the cell cycle. Mol Microbiol *50*, 845-855.

Müller, P., Ewers, C., Bertsche, U., Anstett, M., Kallis, T., Breukink, E., Fraipont, C., Terrak, M., Nguyen-Disteche, M., and Vollmer, W. (2007). The essential cell division protein FtsN interacts with the murein (peptidoglycan) synthase PBP1B in *Escherichia coli*. J Biol Chem *282*, 36394-36402.

Potluri, L., Karczmarek, A., Verheul, J., Piette, A., Wilkin, J.M., Werth, N., Banzhaf, M., Vollmer, W., Young, K.D., Nguyen-Disteche, M., *et al.* (2010). Septal and lateral wall localization of PBP5, the major D,D-carboxypeptidase of *Escherichia coli,* requires substrate recognition and membrane attachment. Mol Microbiol.

Scheffers, D.J., Jones, L.J., and Errington, J. (2004). Several distinct localization patterns for penicillin-binding proteins in *Bacillus subtilis*. Mol Microbiol *51*, 749-764.

Taschner, P.E., Huls, P.G., Pas, E., and Woldringh, C.L. (1988). Division behavior and shape changes in isogenic ftsZ, ftsQ, ftsA, pbpB, and ftsE cell division mutants of *Escherichia coli* during temperature shift experiments. J Bacteriol *170*, 1533-1540.

Typas, A., Nichols, R.J., Siegele, D.A., Shales, M., Collins, S.R., Lim, B., Braberg, H., Yamamoto, N., Takeuchi, R., Wanner, B.L., *et al.* (2008). High-throughput, quantitative analyses of genetic interactions in *E. coli*. Nat Methods *5*, 781-787.

Ursinus, A., van den Ent, F., Brechtel, S., de Pedro, M., Höltje, J.-V., Löwe, J., and Vollmer, W. (2004). Murein (peptidoglycan) binding property of the essential cell division protein FtsN from *Escherichia coli*. J Bacteriol *186*, 6728-6737.

Vollmer, W., von Rechenberg, M., and Höltje, J.-V. (1999). Demonstration of molecular interactions between the murein polymerase PBP1B, the lytic transglycosylase MltA, and the scaffolding protein MipA of *Escherichia coli*. J Biol Chem *274*, 6726-6734.

von Rechenberg, M., Blake, B.K., Ho, Y.S., Zhen, Y., Chepanoske, C.L., Richardson, B.E., Xu, N., and Kery, V. (2005). Ampicillin/penicillin-binding protein interactions as a model drug-target system to optimize affinity pull-down and mass spectrometric strategies for target and pathway identification. Proteomics *5*, 1764-1773.

Werner, J.N., Chen, E.Y., Guberman, J.M., Zippilli, A.R., Irgon, J.J., and Gitai, Z. (2009). Quantitative genome-scale analysis of protein localization in an asymmetric bacterium. Proc Natl Acad Sci U S A *106*, 7858-7863.

Yamamoto, N., Nakahigashi, K., Nakamichi, T., Yoshino, M., Takai, Y., Touda, Y., Furubayashi, A., Kinjyo, S., Dose, H., Hasegawa, M., *et al.* (2009). Update on the Keio collection of *Escherichia coli* single-gene deletion mutants. Mol Syst Biol *5*, 335.
