# Supplementary figures and images for: Regulation of peptidoglycan synthesis by outer membrane proteins
Source: Cell. Author manuscript; Available in PMC 2011 Dec 23. (PMC3060616; doi:10.1016/j.cell.2010.11.038)

Figure S1

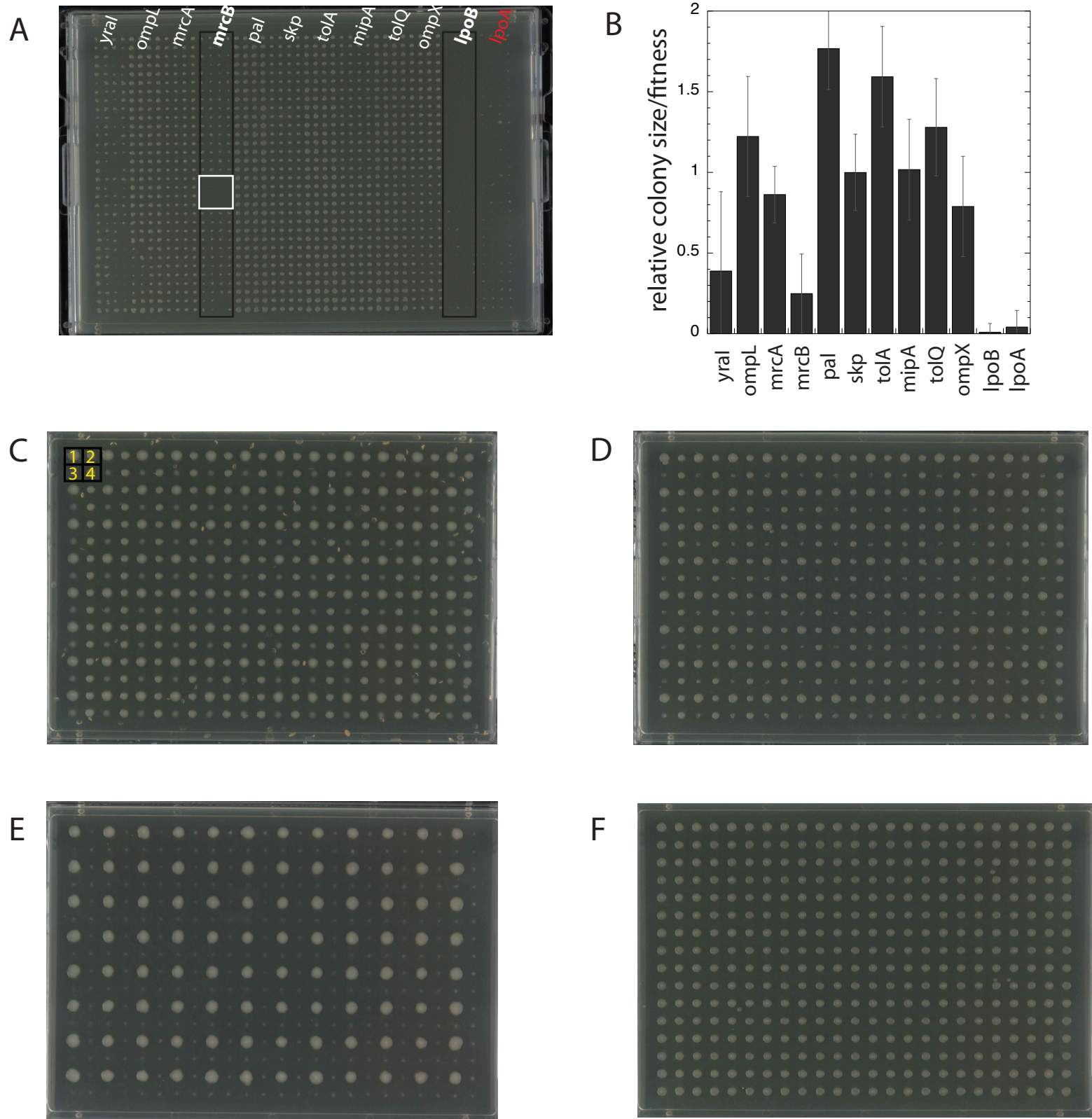

Figure S2

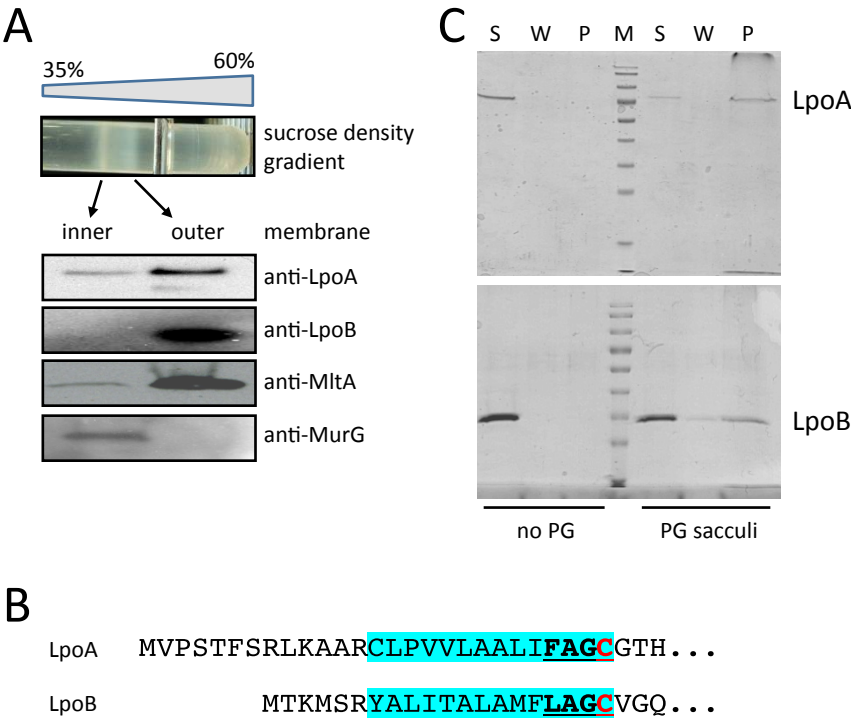

Figure S3

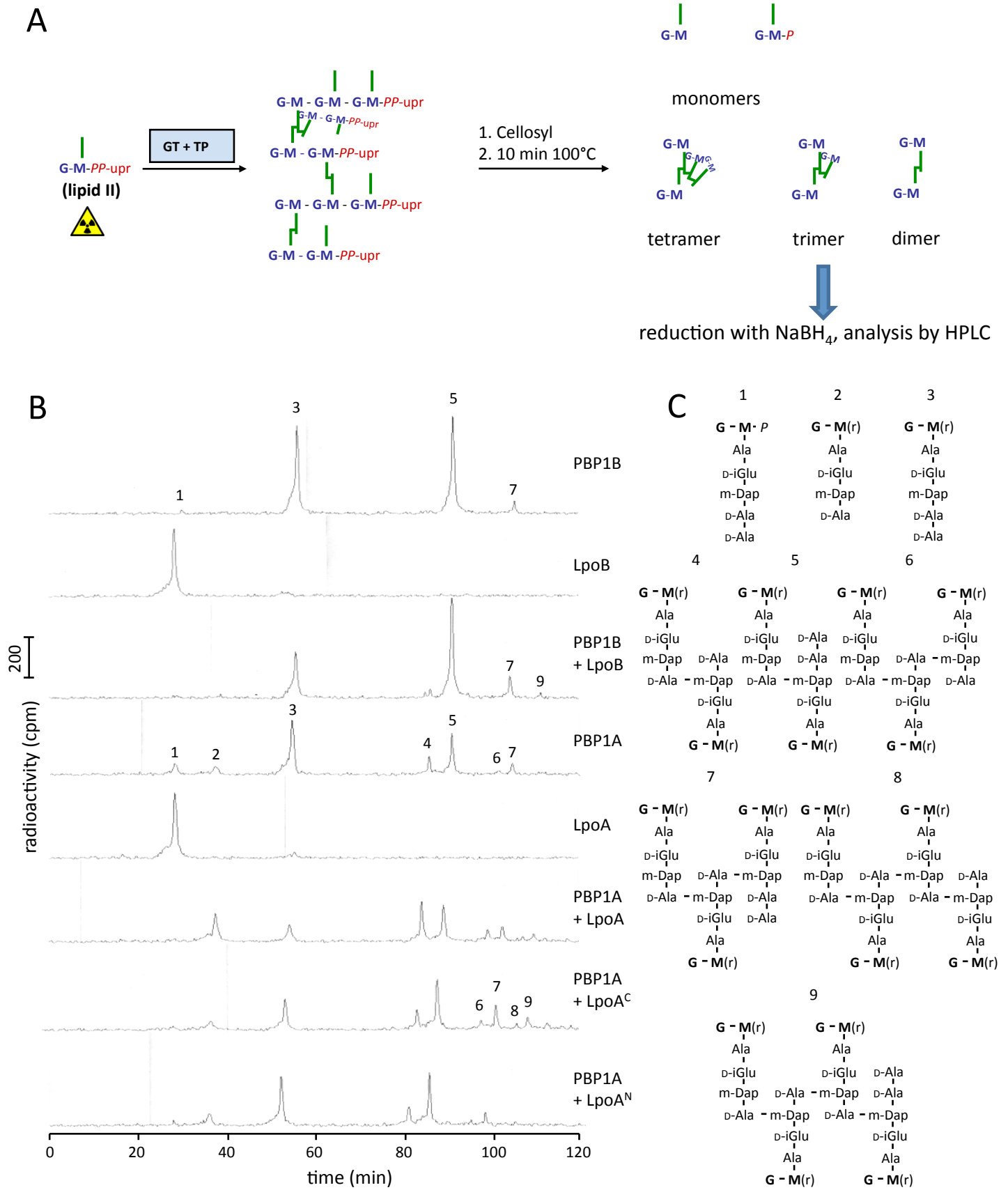

Figure S4

A

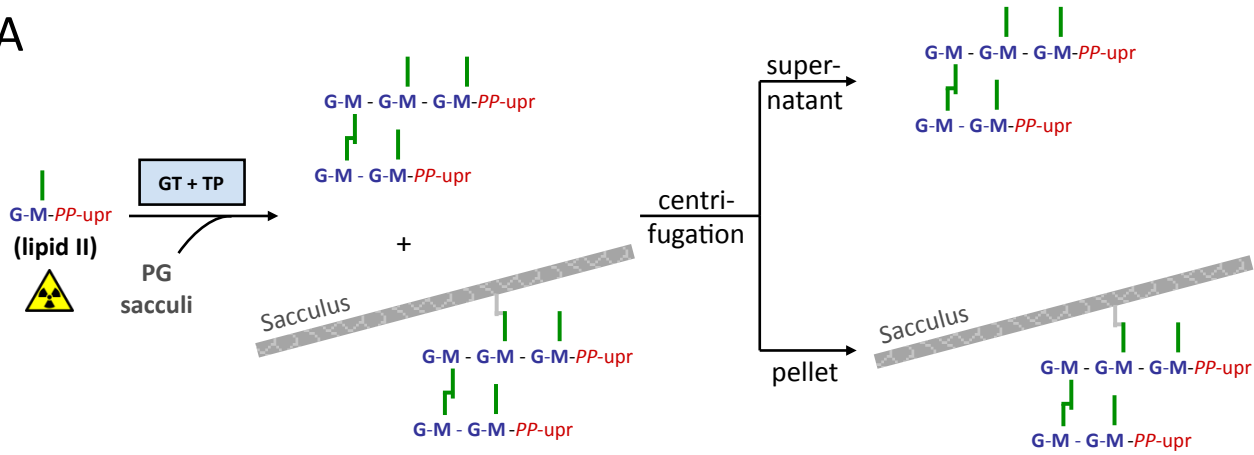

B

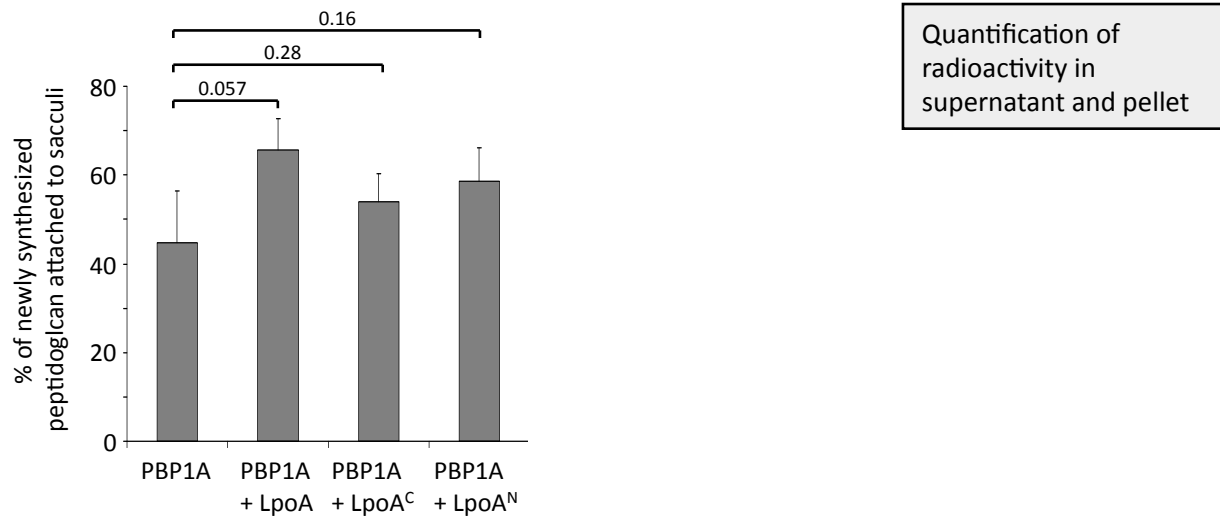

Figure S5

A

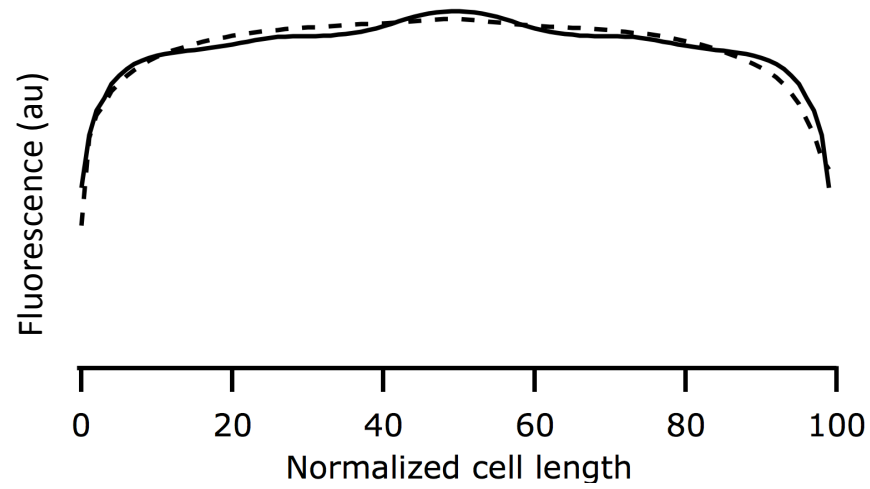

B

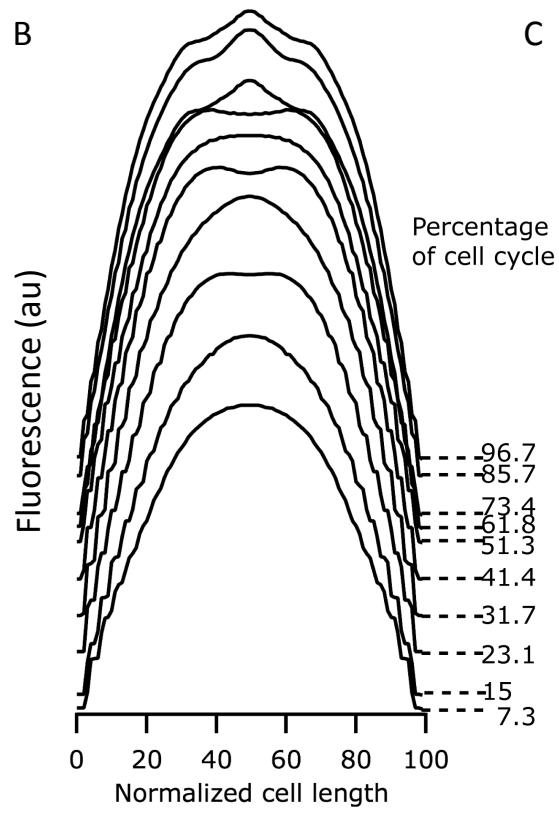

C

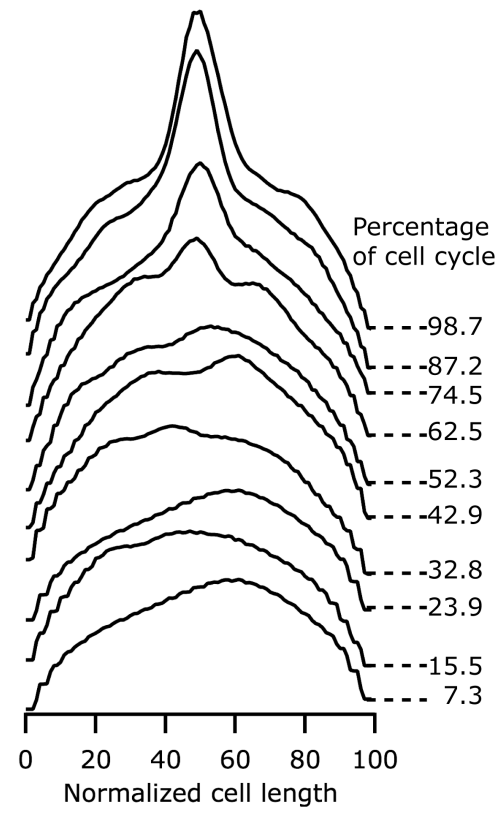

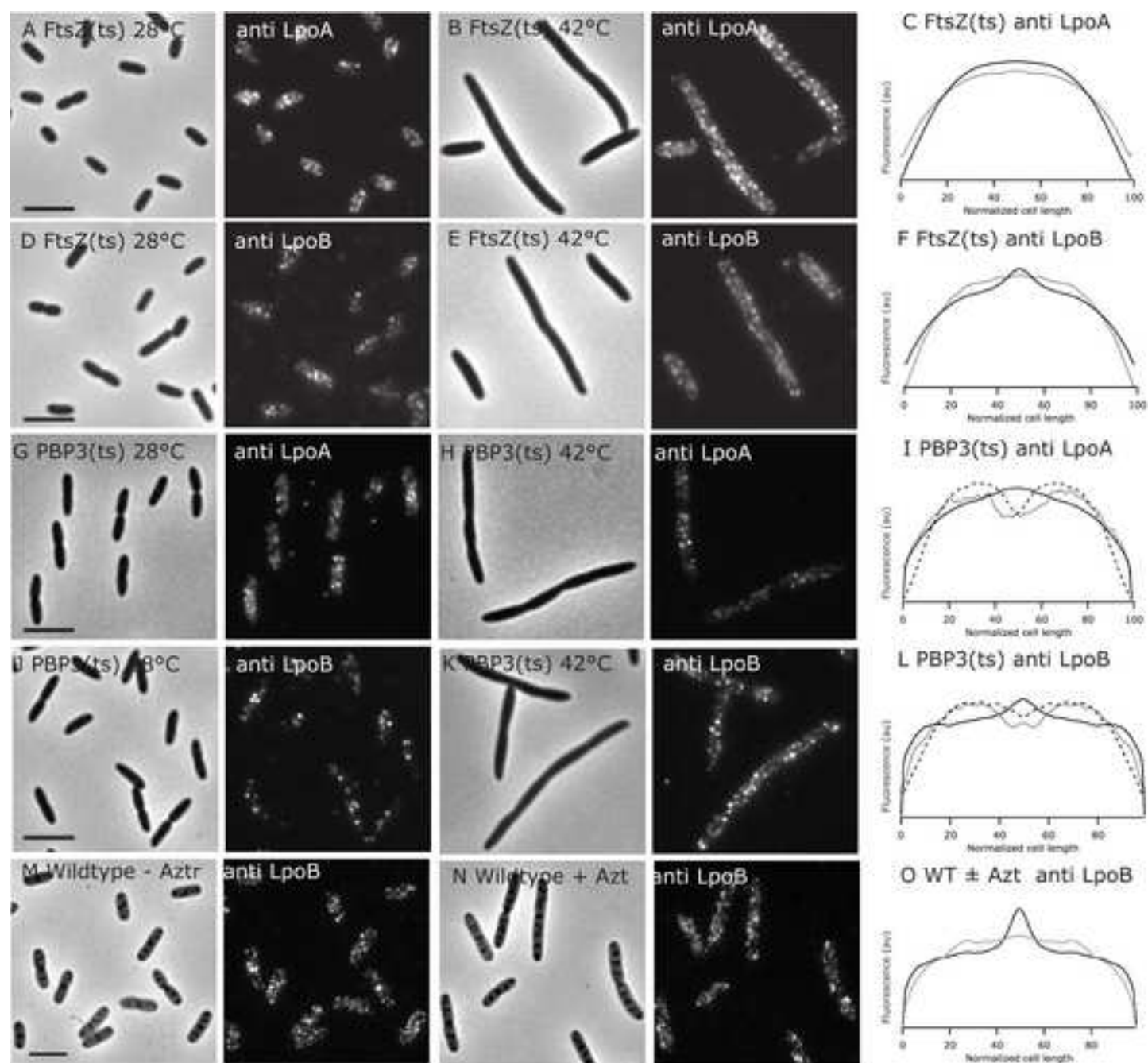

Figure S7

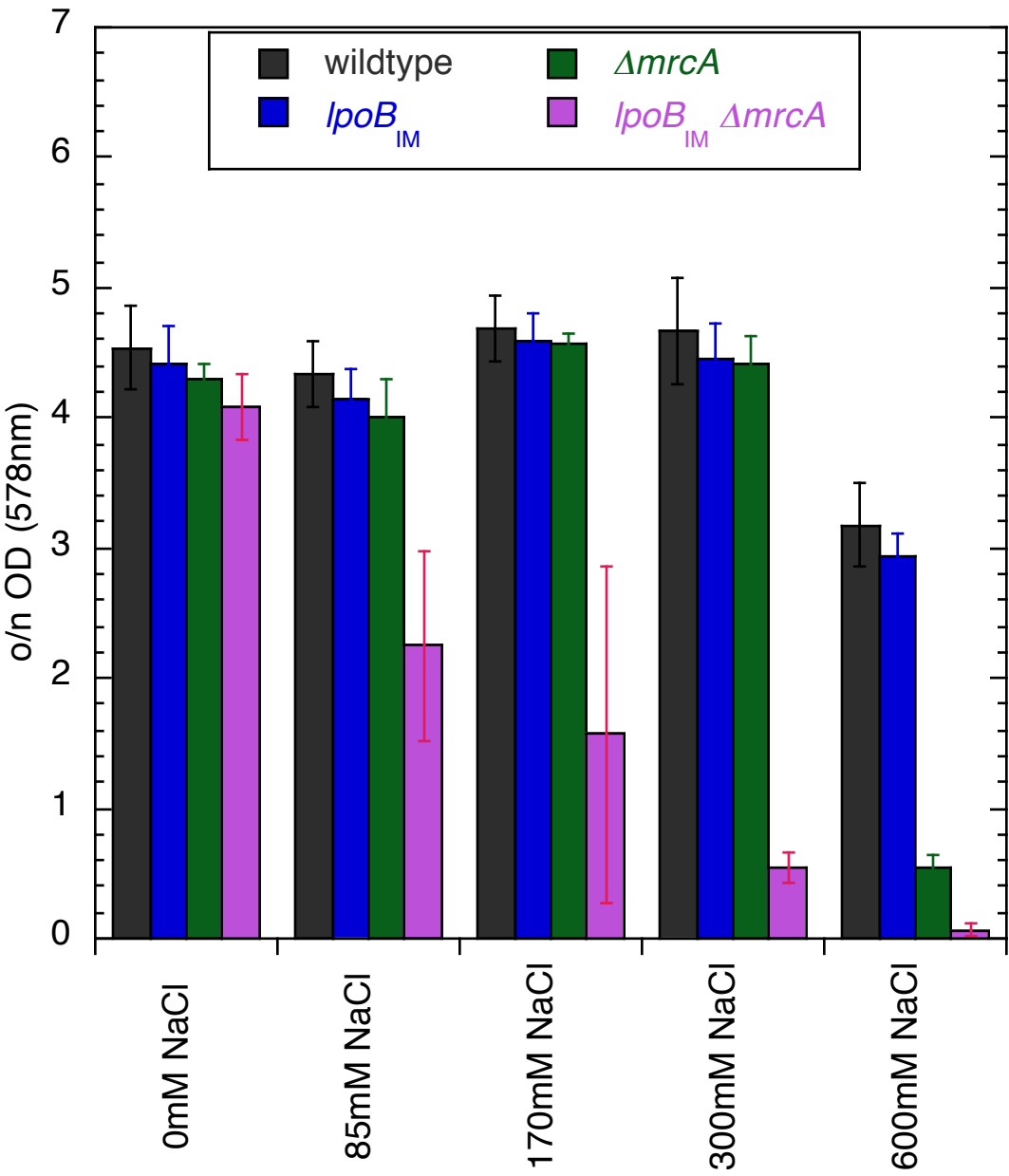

Supplement: 02 [file NIHMS263303-supplement-02.pdf]
